# Regulation of CXCR4 function by S1P_1_ through heteromerization

**DOI:** 10.1186/s12964-025-02099-x

**Published:** 2025-02-26

**Authors:** Hyun-Tae Kim, Jae-Yeon Jeong, Won-Ki Huh

**Affiliations:** 1 School of Biological Sciences, Seoul National University, Seoul, 08826 Republic of Korea; 2 GPCR Therapeutics Inc, Gwanak-gu, Seoul, 08790 Republic of Korea; 3 Institute of Microbiology, Seoul National University, Seoul, 08826 Republic of Korea

**Keywords:** CXCR4, G protein-coupled receptor, Heteromer, S1P_1_

## Abstract

**Background:**

The trafficking of immune cells between lymphoid organs and circulation depends on gradients of CXCL12 and sphingosine-1-phosphate (S1P), mediated through their cognate receptors C-X-C chemokine receptor type 4 (CXCR4) and S1P receptor type 1 (S1P_1_). S1P_1_ facilitates the egress of hematopoietic stem cells and lymphocytes by counteracting CXCR4-mediated retention signals. However, the molecular mechanisms underlying this interplay remain poorly understood. In this study, we uncover CXCR4-S1P_1_ heteromerization and explore their functional interactions.

**Methods:**

Bimolecular fluorescence complementation (BiFC) assay, proximity ligation assay (PLA), and quantitative bioluminescence resonance energy transfer (BRET) assay were employed to detect CXCR4-S1P_1_ heteromerization. Functional properties of the heteromers were assessed using cAMP assay, G protein activation, β-arrestin recruitment, ligand binding, calcium mobilization, and transwell migration assays. S1P_1_-overexpressing Jurkat T cells were generated via lentiviral transduction, while S1P_1_-deficient KARPAS299 cells and β-arrestin1/2-deficient HEK293A cells were constructed using the CRISPR/Cas9 system.

**Results:**

CXCR4-S1P_1_ heteromerization was observed in HEK293A cells overexpressing both receptors. The S1P/S1P_1_ axis interfered with CXCR4-mediated signaling, while CXCR4 did not affect S1P_1_-mediated signaling, indicating a unidirectional modulation of CXCR4 by S1P_1_. CXCL12 binding to CXCR4 remained unchanged in the presence of S1P_1_, and interference of CXCL12-induced Gα_i_ activation by S1P_1_ was observed in β-arrestin1/2-deficient cells. BRET analysis revealed that S1P_1_ interfered with CXCR4-Gα_i_ pre-association and CXCR4 oligomerization, both of which are critical for CXCR4 function. Domain-swapping experiments identified transmembrane domain 3 of S1P_1_ as essential for this modulation. In Jurkat T cells overexpressing S1P_1_, CXCR4-mediated signaling and cell migration were diminished, whereas these functions were enhanced in S1P_1_-deficient KARPAS299 cells. Co-activation of S1P_1_ attenuated CXCL12-induced migration, while pretreatment with S1P or FTY720-phosphate increased CXCR4-mediated migration by downregulating surface S1P_1_ in KARPAS299 cells. In primary T cells, PLA confirmed CXCR4-S1P_1_ heteromerization, and S1P interfered with CXCL12-induced migration.

**Conclusions:**

This study identifies CXCR4-S1P_1_ heteromers and demonstrates a unidirectional modulation of CXCR4 by S1P_1_. S1P_1_ affects CXCR4 function by disrupting its G protein pre-association and oligomerization. These findings underscore the regulatory role of the S1P/S1P_1_ axis in CXCR4 signaling within the heteromeric context and provide novel insights into the intricate mechanisms governing immune cell trafficking.

**Supplementary Information:**

The online version contains supplementary material available at 10.1186/s12964-025-02099-x.

## Background

G protein-coupled receptors (GPCRs) are the largest family of plasma membrane proteins, characterized by seven transmembrane domains (TMs) that mediate intracellular signaling in response to diverse stimuli, such as light, neurotransmitters, lipid mediators, chemokines, and odorant molecules [1, 2]. Ligand binding induces conformational changes in the GPCR, leading to the dissociation of the coupled G protein subunits [3–5]. The human genome encodes sixteen Gα, five Gβ, and twelve Gγ subunits, providing spatial and temporal regulation of GPCR signaling [6]. Once dissociated, these G protein subunits propagate downstream signaling cascades, ultimately regulating a wide array of cellular and physiological responses [7, 8]. In addition to G protein-dependent signaling, GPCRs activate G protein-independent signaling through β-arrestins [9, 10]. These β-arrestins not only mediate receptor desensitization and internalization, but also facilitate sustained endosomal signaling in conjunction with G proteins following GPCR internalization [11–13].

With approximately 800 GPCRs identified in humans, these receptors have gained significant attention due to their essential roles in numerous physiological processes. Over 30% of FDA-approved drugs target GPCRs, addressing a range of conditions such as hypertension, inflammatory disease, pain, and obesity [14]. While GPCRs were traditionally considered to function as monomers, growing evidence has revealed the existence of GPCR heteromers [15]. These heteromers exhibit distinct properties in ligand binding, downstream signaling, and cellular responses compared to their homomers. For instance, the β2-adrenergic receptor (β2AR) facilitates lymphocyte retention in lymph nodes (LNs) through physical interactions with chemokine receptors CXCR4 and CCR7 [16]. Similarly, the lysophosphatidic acid receptor 1 (LPA_1_) regulates lymphatic endothelial junctions by promoting sphingosine-1-phosphate receptor 1 (S1P_1_)/β-arrestin coupling [17]. Using a high-throughput screening approach based on a bimolecular fluorescence complementation (BiFC) assay, we previously identified several GPCR heteromers, including CXCR4-β2AR [18, 19], CXCR4-histamine receptor 1 [20], and CXCR4-LPA_1_ [21].

CXCR4 is a widely expressed chemokine receptor critical for hematopoietic stem/progenitor cells (HSPCs) retention in the bone marrow (BM) and immune cell trafficking between secondary lymphoid organs and peripheral tissues [22–24]. Clinically, CXCR4 inhibitors are used to mobilize HSPCs for transplantation and enhance the circulation of neutrophils and lymphocytes [25–27]. Similarly, lymphocyte retention in LNs is mediated by CXCR4 with their cognate ligands CXCL12 [16, 28, 29]. Furthermore, CXCR4 is overexpressed in various cancers, promoting tumor growth, invasion, metastasis, angiogenesis, and therapeutic resistance [30]. Additionally, CXCR4 blockade has been shown to enhance the efficacy of immune checkpoint inhibitors in pancreatic and colorectal cancers by promoting T cell infiltration into tumors [31–33].

CXCR4 exists in various structural states, including monomers, dimers, and oligomers, with oligomerization playing an essential role in its function [23]. CXCL12 has been shown to induce CXCR4 oligomerization, and studies suggest that higher receptor expression levels correlate with enhanced oligomerization and increased basal activity [18, 34]. Conversely, inverse agonists have been demonstrated to disrupt oligomers, thereby inhibiting CXCR4 activity [35]. Beyond homomeric interactions, CXCR4 forms functional complexes with other surface molecules, such as CCR5, CXCR7, and CD4, further modulating its signaling and functional properties [34, 36]. Interestingly, CD4 coexpression has been reported to impair CXCL12-induced CXCR4 nanoclustering, which results in diminished CXCR4-mediated signaling and migration in Jurkat cells [34].

Sphingosine-1-phosphate (S1P) is a bioactive lipid that regulates cell migration, adhesion, survival, and proliferation through its five receptors S1P_1_ to S1P_5_ [37]. S1P_1_ is ubiquitously expressed, with the highest levels found in endothelial cells and lymphocytes [38, 39]. In endothelial cells, S1P_1_ is essential for cardiovascular development and immune cell egress from lymphoid tissues [38, 40, 41]. Drugs targeting S1P_1_, such as those for multiple sclerosis, prevent lymphocyte egress from lymphoid tissues, thereby reducing autoreactive lymphocyte infiltration into the central nervous system [42]. Additionally, S1P_1_ contributes to cancer progression by promoting cancer cell migration and survival and enhancing regulatory T cell accumulation [43, 44]. While CXCR4 and S1P_1_ are coexpressed in cancer and immune cells and participate in overlapping processes, their interaction remains complex. CXCR4 mediates HSPC retention in the BM, counteracted by the S1P gradient driving HSPC migration toward circulation [45, 46]. Interestingly, S1P_1_ expression is essential for CXCR4 antagonist-induced HSPC mobilization, further highlighting their interplay [45], while the S1P_1_ agonist FTY720 enhances CXCR4-mediated HSPC homing to the BM [47]. Such opposing effects underscore the complexity of CXCR4-S1P_1_ interactions, necessitating further study.

In this study, we identified and validated the heteromerization between CXCR4 and S1P_1_ and demonstrated how the S1P/S1P_1_ axis modulates CXCR4 function by sequestering G proteins and disrupting oligomerization. Additionally, we observed that downregulation of S1P_1_ by FTY720-phosphate (FTY720P) enhanced CXCL12-induced migration. Our findings provide novel insights into the molecular mechanisms governing CXCR4-S1P_1_ interactions and their implications for immune cell trafficking.

## Methods

### Plasmids and cloning

Human cDNAs for *CXCR4*, *S1PR1*, *S1PR3*, *OPRM1*, *GNAI1*, *GNAI2*, *GNAI3*, *GNAOA*, *GNAOB*, *GNB1*, and *GNG2* were obtained from the Missouri S&T cDNA Resource Center. These cDNAs were cloned into pENTR201 or pENTR/D-TOPO vectors (Invitrogen) as described previously [48]. If necessary, site-directed mutagenesis was employed to introduce a stop codon at the end of the gene of interest (GOI). The N-terminal fragment of Venus (VN) and the C-terminal fragment of Venus (VC) were derived from the AdBiFC vectors [49]. Plasmids with Flag, HA, and MYC tags at the N-terminus of each GPCR were generated using one-step sequence- and ligation-independent cloning (SLIC) [50]. pcDNA3.1-Rluc8 vectors were kindly provided by Hee-Jung Choi [51], and pLenti6/V5-DEST Gateway vector was purchased from Invitrogen. pcDNA3.1 destination vectors with C-terminal VN, VC, Rluc8 and mCitrine (mCit) tags were constructed using SLIC. Subsequently, GOIs were cloned into pcDNA3.1 destination vectors using the Gateway cloning system according to the manufacturer’s instructions (Invitrogen). For CRISPR/Cas9-mediated genome editing, a control guide RNA (sgControl, 5′-ACGGAGGCTAAGCGTCGCAA-3′), a guide RNA targeting human *ARRB1* (sg*ARRB1*, 5′-TTCCCCGTGTCTTCGGGCCC-3′; human *ARRB2* (sg*ARRB2*, 5′-GCGGGACTTCGTAGATCACC-3′), and human *S1PR1* (sg*S1PR1*, 5′-CACCGGACGCTCAGGACGATAATTA-3′) were cloned into lentiCRISPR-v2, a plasmid kindly provided by Feng Zhang (Addgene, #52961). Domain-swapped mutant forms of S1P_1_/S1P_3_ were constructed using SLIC of each insert into the inverse PCR product of the backbone vectors. The sequences of TM3 of S1P_1_ (KLTPAQWFLREGSMFVALSASVFSLLAIAIERYITMLKMKLHNG), TM4 of S1P_1_ (SNNFRLFLLISACWVISLILGGLPIMGWNCISALSSCS), TM3 of S1P_3_ (SLSPTVWFLREGSMFVALGASTCSLLAIAIERHLTMIKMRPYDA), and TM4 of S1P_3_ (NKRHRVFLLIGMCWLIAFTLGALPILGWNCLHNLPDCS) were based on previous reports [17, 52] and structural information (PDB: 3V2Y and 7C4S) [53, 54].

### Cell culture

HEK293A, Jurkat clone E6-1, and KARPAS299 cells were acquired from the Invitrogen, Korean Cell Line Bank, and the European Collection of Authenticated Cell Cultures, respectively. HEK293A cells were cultured in Dulbecco’s modified Eagle’s medium (DMEM) (HyClone, SH30243.01) supplemented with 10% fetal bovine serum (FBS) (HyClone, SH30084.03). Jurkat and KARPAS299 cells were cultured in Roswell Park Memorial Institute (RPMI) 1640 medium (HyClone, SH30027.01) supplemented with 10% FBS. All cells were cultured at 37 °C in a 5% CO_2_ humidified atmosphere. For transient transfection, HEK293A cells were seeded at a density of 4 × 10^5^ cells per well in 6-well plates or 1 × 10^5^ cells per well in 24-well plates. After 24 h, transient transfection was performed using PEI MAX (1 mg/ml) (Polysciences, #24765). Control Jurkat cells and Jurkat cells overexpressing MYC-tagged S1P_1_ or S1P_3_ were generated by transducing cells with lentivirus encoding empty vector and MYC-S1P_1_ or -S1P_3_, followed by selection with 50 µg/ml blasticidin (InvivoGen). sg*S1PR1* and sgControl KARPAS299 cells were generated by transducing cells with lentivirus encoding CRISPR/Cas9-sg*S1PR1* or CRISPR/Cas9-sgControl, followed by selection with 0.5–2.0 µg/ml puromycin (InvivoGen).

### Reagents and antibodies

CXCL12 (#300–28 A) was purchased from Peprotech. IT1t (#4596), S1P (#1370), and Ex26 (#5833) were purchased from Tocris Bioscience. Forskolin (#HY-15371) and FTY720P (#HY-15382) were purchased from MedChemExpress. Coelenterazine h (#301), coelenterazine 400a (#340), and coelenterazine native (#303) were purchased from Nanolight Technology. Normal rabbit IgG control antibody (#2729), anti-MYC-tag (9B11, #2276), and anti-HA-tag (C29F4, #3724) were purchased from Cell Signaling Technology. Anti-CXCR4 mouse monoclonal antibody (4G10, #sc-53534) was purchased from Santa Cruz Biotechnology. Mouse IgG control antibody (#I-2000) was purchased from Vector Laboratories. Anti-human S1P_1_/EDG-1 mouse monoclonal antibody (#MAB2016), anti-human S1P_1_/EDG-1 AF488-conjugated mouse monoclonal antibody (#FAB2016G), mouse F(ab)2 IgG (H + L) APC-conjugated antibody (#F0101B), and anti-human IgG APC-conjugated antibody (#0135) were purchased from R&D Systems. Alexa Fluor 647 donkey anti-mouse IgG (H + L) (#31571), Goat anti-Rabbit IgG (H + L) secondary Alexa Fluor Plus 488 (#A32731) and Goat anti-Rabbit IgG (H + L) secondary Alexa Fluor Plus 568 (#A11011) were purchased from Invitrogen. PerCP/Cyanine5.5 anti-human CD3 (UCHT1, #300430) and PE anti-human CD4 (RPA-T4, #300508) were purchased from Biolegend. TZ14011-AF488 was synthesized by AnyGen (Korea). Anti-CXCR4 (Ulocuplumab) human monoclonal antibody was synthesized by Genscript [55].

### Bimolecular fluorescence complementation (BiFC) assay and immunocytochemistry

HEK293A cells were seeded in a 6-well plate and transfected with the indicated plasmids. One day after transfection, cells were detached using DPBS containing 2 mM EDTA and centrifuged at 500 *g* for 5 min. Cells were then resuspended in DMEM containing 10% FBS and transferred to a poly-D-lysine-coated 96-well clear bottom black plate (Greiner, #655090). The following day, cells were fixed with 4% paraformaldehyde for 30 min at room temperature, blocked with DPBS containing 0.5% BSA, and stained with anti-CXCR4 mouse monoclonal antibody (4G10, 1:200) and anti-HA-tag rabbit monoclonal antibody (C29F4, 1:2,000) overnight at 4 °C. After washing with DPBS, the samples were incubated with AF647-conjugated mouse secondary antibody (1:1,000) and AF568-conjugated rabbit secondary antibody (1:2,000). Following nuclear staining with Hoechst 33342, images were acquired using an LSM 700 laser scanning confocal microscope (Zeiss) with a 20× objective.

### Proximity ligation assay (PLA)

PLA was performed using the NaveniFlex or NaveniFlex Cell MR Atto647N kits (Navinci) according to the manufacturer’s instructions. In summary, HEK293A cells were seeded in a 6-well plate and transfected with plasmids encoding Flag-CXCR4, HA-S1P_1_, or HA-µOR1, either individually or in combination using PEI MAX (1 mg/ml) (Polysciences, #24765). 24 h post-transfection, cells were detached with DPBS containing 2 mM EDTA and centrifuged at 500 *g* for 5 min. The cell pellet was resuspended in DMEM containing 10% FBS and transferred to a poly-D-lysine-coated 96-well clear bottom black plate (Corning, #3340). The following day, cells were fixed with 4% paraformaldehyde for 30 min at room temperature and blocked with the kit’s blocking solution. For single PLA, cells were stained with either an anti-CXCR4 mouse monoclonal antibody (Santa Cruz Biotechnology, #sc-53534, 4G10, 1:2,000) or an anti-HA-tag rabbit monoclonal antibody (Cell Signaling Technology, #3724, C29F4, 1:10,000) overnight at 4 °C. This was followed by incubation with anti-mouse probes or anti-rabbit probes, respectively, to detect GPCR homomers. For double PLA, cells were stained with both anti-CXCR4 and anti-HA-tag antibodies, followed by incubation with anti-mouse and anti-rabbit probes together to detect GPCR heteromers. The PLA signal was visualized using Atto647N red fluorescent dye. Following nuclear staining with Hoechst 33342, images were acquired using an LSM 700 laser scanning confocal microscope (Zeiss) with a 20× objective for quantification. For KARPAS299 cells and primary T cells, 2 × 10^5^ cells were seeded onto slide glasses using a Cytospin at 500 *g* for 5 min. Cells were stained with probe-conjugated anti-CXCR4 human monoclonal antibody (Ulocuplumab, 10 µg/ml for KARPAS299 cells, 50 µg/ml for primary T cells) and an anti-S1P_1_ mouse monoclonal antibody (R&D systems, #MAB2016, clone 218713, 5 µg/ml) followed by anti-mouse probes. Ulocuplumab was synthesized by Genscript. Human IgG control antibody (#C0045-3) was purchased from Medical & Biological Laboratories. Probe conjugation was performed by Navinci. Three images per sample were acquired, and the area of PLA signal and the numbers of nuclei were quantified using ImageJ software [56].

### Quantitative BRET analysis

To study the interaction between CXCR4 and S1P_1_ or µOR1, fixed amounts of CXCR4-Rluc8, S1P_1_-Rluc8, or µOR1-Rluc8 and increasing amounts of CXCR4-mCit were used for transfection. For domain-swapped mutants, a fixed amount of CXCR4-Rluc8 and increasing amounts of mCit-tagged S1P_1_, S1P_3_, and their domain-swapped mutants were used for transfection. Two days after transfection, cells were detached using DPBS containing 2 mM EDTA, centrifuged at 500 *g* for 5 min, and resuspended in HBSS containing 20 mM HEPES and 0.1% BSA. Subsequently, cells were seeded into a 96-well white plate (Corning, #3917, or SPL, #34296) for measuring BRET signals and luminescence, and a 96-well clear bottom black plate (Corning, #3340) for measuring fluorescence. Coelenterazine h was added at a final concentration of 5 µM. For BRET signals and luminescence measurements, cells were read using a TriStar^2^ LB 942 multimode microplate reader with a 480 nm filter for Rluc8 and a 540 nm filter for mCit, with a detection time of 0.1 s per well. For fluorescence measurements, cells were read using a TriStar^2^ LB 942 multimode microplate reader with a 485 nm excitation filter and a 535 nm emission filter, with a detection time of 0.2 s per well. The Net BRET ratio was calculated by subtracting the BRET ratio of donor (Rluc8)-only-expressed sample from that of donor (Rluc8)-acceptor (mCit)-coexpressed sample. Saturation curves were analyzed through non-linear regression using GraphPad Prism 5 Software. BRET_max_ and BRET_50_ values were derived from the fitted saturation curves.

### Flow cytometry

HEK293A cells were detached using Accutase (Sigma-Aldrich, #A6964). Cells were stained with indicated antibodies for 1 h on ice. Subsequently, cells were labeled with APC-conjugated anti-mouse, APC-conjugated anti-human, and AF488-conjugated anti-rabbit secondary antibodies. HEK293A, Jurkat, and KARPAS299 cells were analyzed using a LSRFortessa X-20 flow cytometer (BD Biosciences) and primary T cells were analyzed using a Canto II flow cytometer (BD biosciences). Flow cytometry data were processed with FlowJo software, and relative cell surface expression of GPCRs was expressed as mean fluorescence intensity (MFI).

### GloSensor cAMP assay

Quantification of cAMP production was conducted using the GloSensor cAMP assay (Promega, #E1291). HEK293A cells were seeded in 6-well plates and transfected with plasmids encoding GloSensor-22 F, a biosensor variant with cAMP binding domain fused to a mutant form of luciferase [57], and GPCRs. One day after transfection, cells were detached with DPBS containing 2 mM EDTA and centrifuged at 500 *g* for 5 min. Cells were then resuspended in DMEM without phenol red (Gibco, #21063-029) containing 5% FBS and seeded into a 96-well white plate (Corning, #3917, or SPL, #34296). The following day, cells were washed with assay buffer (CO_2_-independent medium (Gibco, #18045-088) with 0.1% BSA) and incubated in the dark at 37 °C for 2 h with 800 µg/ml D-luciferin. To measure ligand-induced inhibition of cAMP accumulation, cells were stimulated with 3 µM forskolin, followed by agonist treatment. Luminescence was measured using a TriStar^2^ LB 942 multimode microplate reader (Berthold Technologies) with a 540 nm filter and a detection time of 1 s per well for a duration of 20 min.

### G protein activation assay

Using TRUPATH system [58], ligand-induced G protein activation was quantified by determining the dissociation between Gα and Gβγ heterotrimers. Plasmids were kindly provided by Bryan Roth (Addgene, Kit #1000000163). GPCRs were cotransfected with the Gα-Rluc8, Gβ, and GFP2-Gγ, each at 40 ng, in a 6-well plate as previously described [58]. One day after transfection, cells were detached using DPBS containing 2 mM EDTA and centrifuged at 500 *g* for 5 min. Cells were then resuspended in DMEM without phenol-red containing 5% FBS and seeded into a 96-well white plate (Corning, #3917, or SPL, #34296). The following day, cells were washed with the assay buffer (HBSS containing 20 mM HEPES and 0.1% BSA) and treated with Coelenterazine 400a (5 µM) and CXCL12 or S1P. Antagonists were pretreated 30 min before agonist treatment. BRET2 signal was measured using a TriStar^2^ LB 942 multimode microplate reader with a 410 nm filter for Rluc8 and a 515 nm filter for GFP2, with a detection time of 0.1 s per well. For ligand-induced G protein recruitment assay and detection of pre-association between GPCRs and G protein, CXCR4-mCit or S1P_1_-mCit, were cotransfected with Gα_i2_-Rluc8, Gβ1, and Gγ2 in a 6-well plate in the presence or absence of MYC-S1P_1_ or Flag-CXCR4. Each Gα protein containing Rluc8 in the middle of the structure was cloned using SLIC as reported previously [59–61]. Two days after transfection, cells were detached using DPBS containing 2 mM EDTA and centrifuged at 500 *g* for 5 min. Cells were resuspended in the assay buffer and treated with Coelenterazine h (5 µM). BRET1 signal was measured using a TriStar^2^ LB 942 multimode microplate reader with a 480 nm filter for Rluc8 and a 540 nm filter for mCit, with a detection time of 0.1 s per well. The BRET ratio was calculated by dividing the long-wavelength emission (GFP2 or mCit signal) by the short-wavelength emission (Rluc8 signal). The Net BRET ratio was calculated by subtracting the BRET ratio of donor (Rluc8)-only-expressed sample from that of donor (Rluc8)-acceptor (mCit)-coexpressed sample. The ΔBRET ratio was calculated by subtracting the BRET ratio of vehicle-treated sample from that of ligand-treated sample.

### β-arrestin recruitment assay

Ligand-induced β-arrestin recruitment was quantified by measuring the recruitment of β-arrestin to GPCRs using BRET. HEK293A cells were transfected with mCit-β-arrestin2 and CXCR4-Rluc8 or S1P_1_-Rluc8 in the absence or presence of MYC-S1P_1_ or Flag-CXCR4, respectively. One day after transfection, cells were detached using DPBS containing 2 mM EDTA and centrifuged at 500 *g* for 5 min. Cells were then resuspended in DMEM without phenol red containing 5% FBS and seeded into a 96-well white plate. The following day, cells were washed with the assay buffer and treated with the assay buffer containing the appropriate ligand and Coelenterazine h at a final concentration of 5 µM. BRET1 signals were read using a TriStar^2^ LB 942 multimode microplate reader with a 480 nm filter for Rluc8 and a 540 nm filter for mCit, with a detection time of 0.1 s per well.

### CXCR4 ligand binding assay

CXCL12 binding to CXCR4 was analyzed as previously reported [21]. Cells were transfected with Gluc-CXCR4 alone or in combination with MYC-S1P_1_. One day after transfection, cells were detached using DPBS containing 2 mM EDTA and centrifuged at 500 *g* for 5 min. Cells were then resuspended in DMEM without phenol red containing 5% FBS and seeded into a 96-well white plate (Corning, #3917, or SPL, #34296). The following day, cells were washed with the assay buffer (HBSS containing 20 mM HEPES and 0.1% BSA). For the saturation binding assay, either vehicle or IT1t (10 µM) was treated for 30 min for total binding and non-specific binding, respectively. Subsequently, the indicated concentration of TZ14011-AF488 was treated with the assay buffer containing coelenterazine native at a final concentration of 5 µM. For competition binding assay, TZ14011-AF488 (10 nM) was treated for 30 min before the indicated concentration of unlabeled CXCL12. BRET1 signals were measured using a TriStar^2^ LB 942 multimode microplate reader with a 480 nm filter for Gluc and a 540 nm filter for AF488, with a detection time of 0.1 s per well. The BRET ratio was calculated as described above. Saturation and competition curves were analyzed through non-linear regression with GraphPad Prism 5 Software. BRET_max_ and BRET_50_ values were derived from the fitted saturation curves.

### Calcium flux assay

Jurkat cells were seeded in PDL (Sigma, #P1024)-coated 96-well black clear-bottom plates (Corning, #3340) at a density of 1 × 10^5^ cells per well. After 24 h, cells were washed with assay buffer (HBSS without phenol red and with 20 mM HEPES and 0.1% bovine serum albumin) and stained with Cal-520AM (4.44 µg/ml; AAT Bioquest) for 2 h at 37℃. Cells were then washed three times with the assay buffer and incubated for an additional 30 min at 37℃. Plates were loaded into a FlexStation 3 multi-mode microplate reader (Molecular Devices), and ligands were injected. Intracellular calcium mobilization was detected for 130 s with an excitation of 485 nm and an emission of 525 nm. The area under the curve was calculated by GraphPad Prism 5 software.

### Transwell migration assay

Jurkat, KARPAS299, and primary T cells were serum-starved overnight in RPMI 1640 containing 0.1% BSA. Cells were loaded into the upper chambers with 5 μm pore size membrane inserts (Corning, #3421) and allowed to migrate into the lower chambers containing CXCL12 or S1P. After incubation at 37 °C for 6 h, migrated cells were collected from the lower chamber and analyzed using a LSRFortessa X-20 flow cytometer with a flow rate of 2 µl/s for 40 s for Jurkat and KARPAS299 cells, and a Canto II flow cytometer with a flow rate of 1 µl/s for 30 s for primary T cells.

### Primary T cell isolation

Human peripheral blood samples were obtained as leukocyte reduction system chambers from the Korean Redcross Blood Services with approval from the Institutional Review Board of Seoul National University (IRB No. E2212/003–003). Peripheral blood mononuclear cells (PBMCs) were isolated through a density gradient centrifugation procedure using Histopaque (Sigma-Aldrich, #10771) and SepMate PBMC isolation tubes (STEMCELL Technologies, #86450). Primary CD3 + CD4 + T cells were isolated using EasySep Human CD4 + T cell negative selection kit (STEMCELL Technologies, #17952), according to the manufacturer’s instructions. The population and expression characteristics of PBMCs and CD4 + T cells were analyzed using anti-CD3 (UCHT1) and anti-CD4 (RPA-T4) antibodies. Cells were cultured in RPMI 1640 medium supplemented with 10% FBS and 50 IU of interleukin-2 (Biolegend, #589104).

### Statistical analysis

Data are expressed as the mean ± SEM or mean ± SD from *n* independent experiments that were performed in duplicate or triplicate for each experiment on different days. Data were analyzed using GraphPad Prism 5 software. Statistical analysis was performed as described in the figure legends.

## Results

### Identification and validation of CXCR4-S1P_1_ heteromerization

Using a high-throughput screen based on a BiFC assay [48], we identified several GPCR heteromers, including CXCR4-β2AR [18, 19], CXCR4-histamine receptor 1 [20], CXCR4-LPA_1_ [21], CXCR4-S1P_1_, and CXCR4-S1P_3_. While S1P_1_ is known to interact with LPA_1_ [17] and CCR5 [62], heteromerization between CXCR4 and S1P_1_ has not, to our knowledge, been previously reported.

**Fig. 1:**
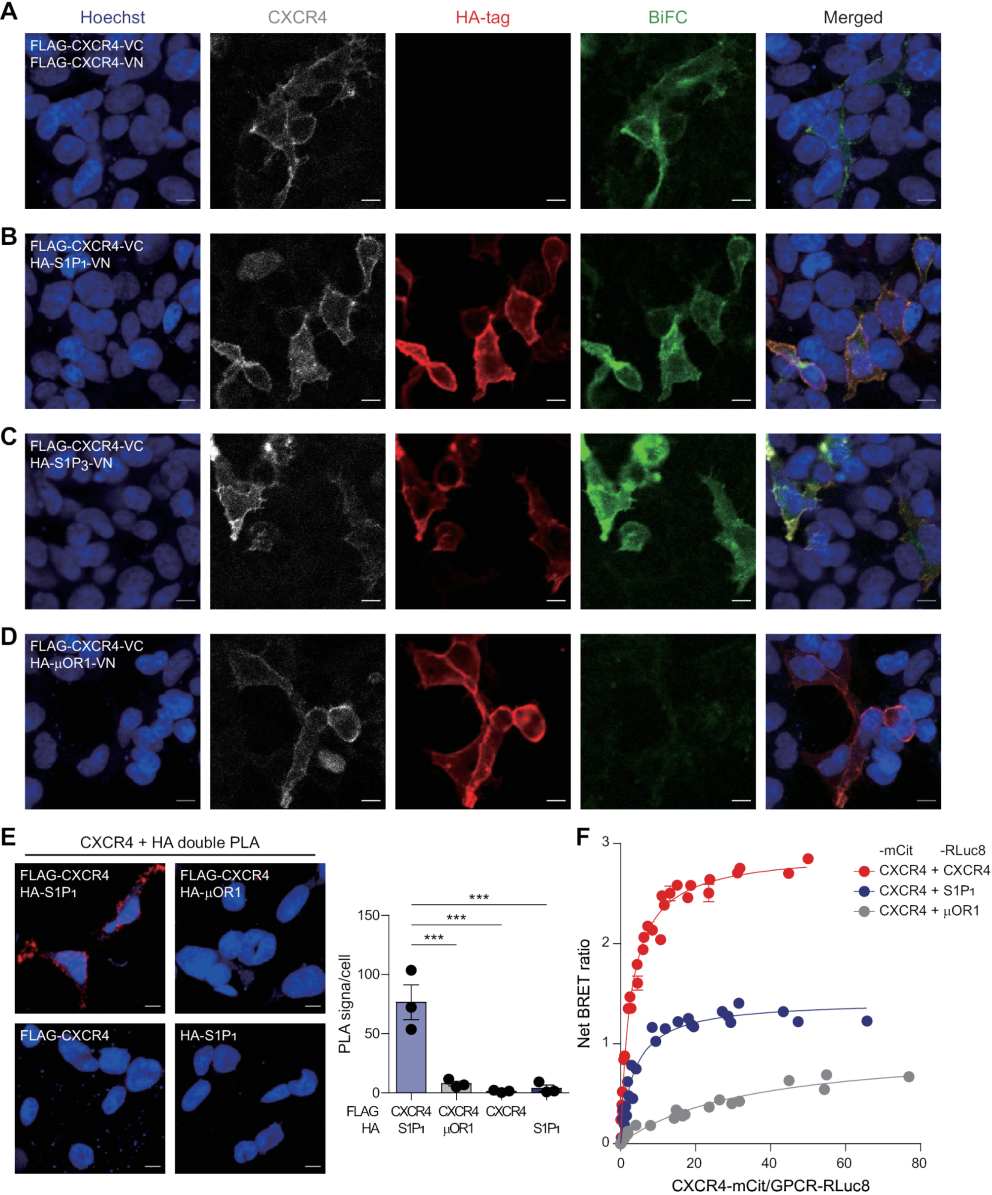
Detection of heteromerization between CXCR4 and S1P1 in HEK293A cells. (A-D) BiFC assay was conducted to detect GPCR interactions in HEK293A cells. Cells were transfected with Flag-CXCR4-VC in combination with VN-tagged Flag-CXCR4 (A), HA-S1P1 (B), HA-S1P3 (C), and HA-µOR1 (D). Surface expression of GPCRs was visualized by staining cells with anti-CXCR4 (4G10) and anti-HA-tag (C29F4) antibodies. Cells were visualized using Hoechst 33342 (blue) for nuclei, AF647 (gray) for CXCR4, AF568 (red) for HA-tagged GPCRs (S1P1, S1P3, and µOR1), and BiFC signals (green). Scale bars: 10 μm. (E) PLA was performed to visualize the proximity between GPCRs in HEK293A cells expressing Flag-CXCR4 and HA-S1P1, Flag-CXCR4 and HA-µOR1, or either receptor alone using anti-CXCR4 (4G10) and anti-HA-tag (C29F4) antibodies. PLA signals were shown in red and cells were visualized using Hoechst 33342 (blue) for nuclei. Images are representative of three independent experiments. Data represent the mean ± SEM of *n* = 3 independent experiments. Statistical significance was tested using one-way ANOVA followed by Tukey’s post-hoc test. ***P < 0.001. Scale bars: 10 μm. (F) Quantitative BRET assay was performed in HEK293A cells between CXCR4-mCit and Rluc8-tagged CXCR4, S1P1, or µOR1. The curves were fitted using a non-linear regression equation and represent three independent experiments

In HEK293A cells overexpressing CXCR4-VN and CXCR4-VC, a BiFC signal was observed at the plasma membrane, indicating CXCR4 homomerization (Fig. 1A). Similarly, BiFC signals were detected in cells coexpressing CXCR4-VC and S1P_1_-VN (Fig. 1B) as well as CXCR4-VC and S1P_3_-VN (Fig. 1C). In contrast, no BiFC signal was observed in cells coexpressing CXCR4-VC and µOR1-VN, despite µOR1-VN being robustly expressed on the surface (Fig. 1D), comparable to CXCR4, S1P_1_, and S1P_3_ in non-permeabilized cells. These findings suggest the specific formation of CXCR4-S1P_1_ and CXCR4-S1P_3_ heteromers in cells overexpressing these GPCRs, while confirming the absence of interaction between CXCR4 and µOR1, consistent with previous reports [21, 63].

To verify the interaction between CXCR4 and S1P_1_, we performed PLA, a highly sensitive and specific method for detecting protein interactions [64]. First, we confirmed the expression of Flag-CXCR4, HA-S1P_1_, and HA-µOR1 by staining cells with anti-CXCR4 and anti-HA-tag antibodies under permeabilizing conditions. Immunocytochemistry revealed comparable levels and homogeneous surface expression of CXCR4, S1P_1_, and µOR1 in HEK293A cells (Additional file 1: Fig. S1A). However, single PLA for CXCR4 showed aggregated puncta (Additional file 1: Fig. S1B), likely reflecting its reported tendency to oligomerize [23, 35], whereas discrete dot-like signals were observed for S1P_1_ and µOR1 (Additional file 1: Fig. S1C). This discrepancy arises because single PLA detects only GPCR homodimers and oligomers, not monomers. Moreover, the PLA signal area per cell for CXCR4 was larger than for S1P_1_ and µOR1, supporting the presence of CXCR4 oligomers. Double PLA signals were detected exclusively in cells expressing both Flag-CXCR4 and HA-S1P_1_, confirming their specific interaction (Fig. 1E). No signals were detected in cells expressing Flag-CXCR4 and HA-µOR1 or in cells expressing either Flag-CXCR4 or HA-S1P_1_ alone. These observations suggest the specific interaction between CXCR4 and S1P_1_, ruling out non-selective aggregation due to receptor overexpression. Importantly, the presence of single PLA signals for GPCRs, but the absence of double PLA signals in CXCR4-µOR1 coexpression, indicates that CXCR4-S1P_1_ heteromers form in a highly selective manner.

To further validate heteromerization between CXCR4 and S1P_1_, we conducted a quantitative BRET assay using GPCRs tagged with either the BRET donor Rluc8 or the BRET acceptor mCit in HEK293A cells [65]. Increasing the amounts of CXCR4-mCit with a fixed amount of CXCR4-Rluc8 or S1P_1_-Rluc8 resulted in hyperbolic increases in the BRET ratio (BRET_max_ = 2.917$\:\pm\:$0.0277, BRET_50_ = 2.739$\:\pm\:$0.1077 for CXCR4-CXCR4; BRET_max_ = 1.431$\:\pm\:$0.0247, BRET_50_ = 3.611$\:\pm\:$0.2509 for CXCR4-S1P_1_) (Fig. 1F), suggesting the formation of both CXCR4 homomers and CXCR4-S1P_1_ heteromers. In contrast, increasing the amounts of CXCR4-mCit with a fixed amount of µOR1-Rluc8 resulted in an almost linear increase in the BRET ratio, characterized by a low BRET_max_ and high BRET_50_ (BRET_max_ = 0.9753$\:\pm\:$0.0524, BRET_50_ = 33.820$\:\pm\:$3.6320), indicating non-specific random interactions between these two GPCRs. Together, these results confirm the specific heteromerization of CXCR4 with S1P_1_.

### S1P_1_ modulates CXCR4 activation at the level of G protein activation

**Fig. 2:**
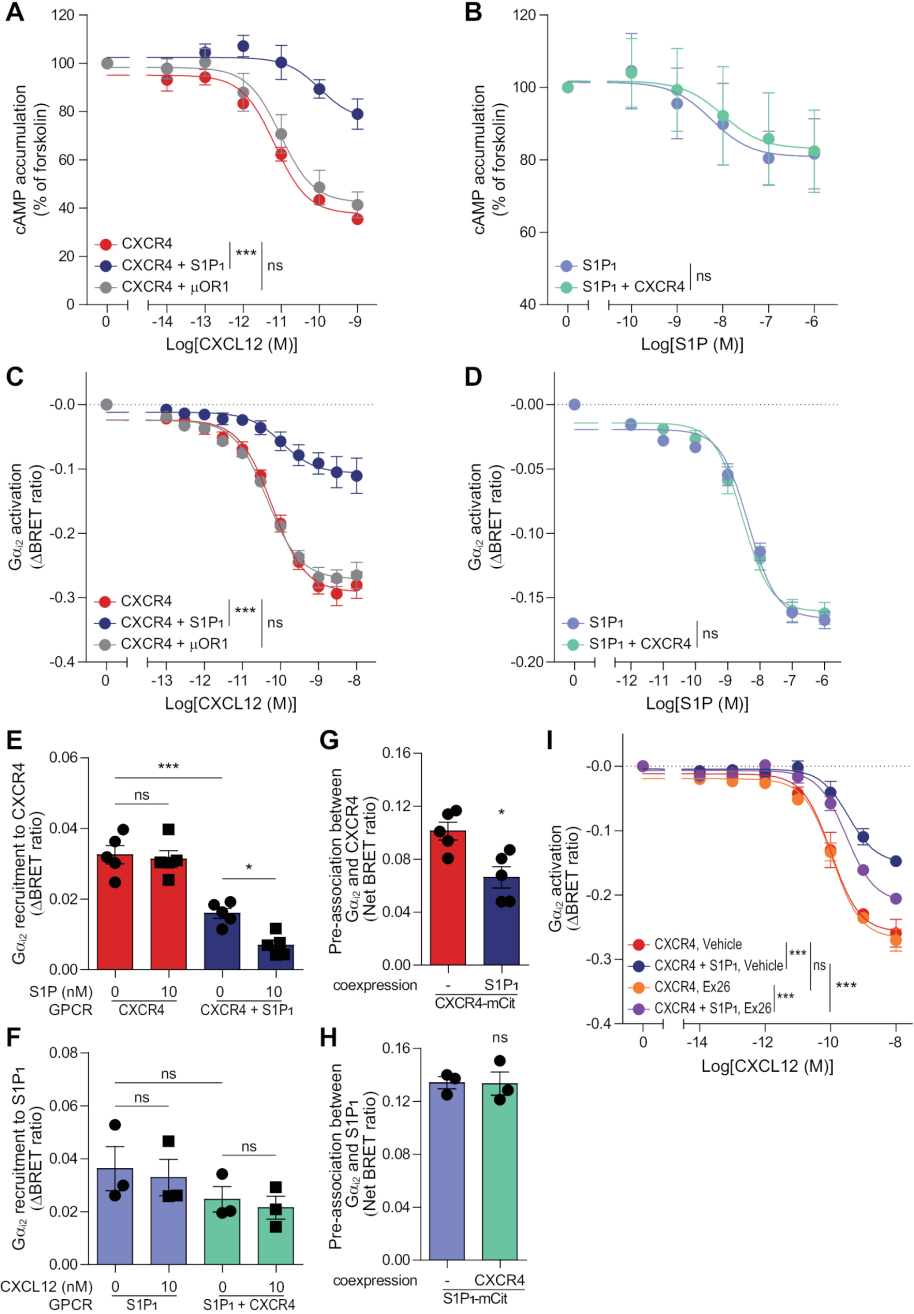
S1P/S1P1 axis interferes with CXCR4-mediated cAMP signaling and Gα activation. (**A**, **B**) CXCL12 (10 nM)- and S1P (10 nM)-induced inhibition of cAMP accumulation was measured using the 22 F cAMP sensor in HEK293A cells expressing CXCR4 alone or together with S1P1 or µOR1 (**A**), and in cells expressing S1P1 alone or together with CXCR4 (**B**). (**C**, **D**) CXCL12 (10 nM)- and S1P (10 nM)-induced Gαi2 activation using Gα-Rluc8, Gβ, and Gγ-GFP2 was measured in HEK293A cells expressing CXCR4 alone or together with S1P1 or µOR1 (**C**), and in cells expressing S1P1 alone or together with CXCR4 (**D**). (**E**,** F**) CXCL12 (10 nM)- and S1P (10 nM)-induced Gαi2 recruitment to CXCR4 and S1P1 using Gα-Rluc8, Gβ, and Gγ was assessed in HEK293A cells expressing CXCR4-mCit alone or together with S1P1 (**E**), and in cells expressing S1P1-mCit alone or together with CXCR4 (**F**). (**G**, **H**) Basal pre-association between GPCR (CXCR4 or S1P1) and Gαi2 was assessed by BRET in HEK293A cells expressing CXCR4-mCit alone or together with S1P1 (**G**) and S1P1-mCit alone or together with CXCR4 (**H**) in the absence of ligand stimulation. (**I**) The effect of S1P1-selective antagonist Ex26 (10 µM) on CXCL12-induced Gαi2 activation was determined in HEK293A cells expressing CXCR4 alone or together with S1P1. Data represent the mean ± SEM of n = 3 to 5 independent experiments. Statistical significance was tested using two-way ANOVA followed by Bonferroni’s post-hoc test (**A**-**D**, **I**), one-way ANOVA followed by Tukey’s post-hoc test (**E**, **F**), and two-tailed Student’s t-test (**G**, **H**). *P < 0.05; ***P < 0.001; ns, not significant

To assess potential influence between GPCR expressions in HEK293A cells, flow cytometry analysis was performed. The coexpression of S1P_1_ or µOR1 did not affect CXCR4 levels (Additional file 1: Fig. S2A). Similarly, the presence of CXCR4 did not alter the surface expression of S1P_1_ and µOR1 (Additional file 1: Fig. S2B). To investigate the effect of S1P_1_ on CXCR4, we monitored CXCR4-mediated cAMP response in HEK293A cells expressing CXCR4 alone or together with S1P_1_ or µOR1. CXCL12 treatment reduced forskolin-induced cAMP accumulation in cells expressing CXCR4 alone (E_max_ = 62.45$\:\pm\:$2.387, LogEC_50_ = -11.17$\:\pm\:$0.1066) (Fig. 2A). Notably, this CXCL12-induced cAMP signaling was significantly counteracted in cells expressing both CXCR4 and S1P_1_ (E_max_ = 23.53$\:\pm\:$6.363, LogEC_50_ = -9.986$\:\pm\:$0.4188). The coexpression of µOR1, a receptor known not to interact with CXCR4, did not alter the CXCL12-induced cAMP signaling (E_max_ = 57.65$\:\pm\:$4.569, LogEC_50_ = -11.02$\:\pm\:$0.1995). Although S1P_1_ is also known as a Gα_i/o_-coupled receptor [66], the coexpression of CXCR4 did not affect the S1P-induced cAMP signaling (Fig. 2B). These findings suggest that S1P_1_ interferes with CXCL12-induced cAMP signaling but not vice versa.

To assess the effect of S1P_1_ on CXCR4-mediated G protein activation, we utilized a BRET-based biosensor, measuring the decrease in the BRET ratio between Gα-Rluc8 and Gγ-GFP2 [58]. Given that Gα_i2_ is prevalent within the Gα_i/o_ subfamily [67], we transfected HEK293A cells with Gα_i2_-Rluc8, Gβ_3_, and Gγ_9_-GFP2. When cells expressing CXCR4 alone were treated with CXCL12, we observed a dose-dependent activation of Gα_i2_ (ΔBRET_max_ = -0.2914$\:\pm\:$0.0071, LogΔBRET_50_ = -10.22$\:\pm\:$0.0673) (Fig. 2C). However, CXCL12-induced Gα_i2_ activation was significantly reduced when S1P_1_ was coexpressed (ΔBRET_max_ = -0.1066$\:\pm\:$0.0084, LogΔBRET_50_ = -9.968$\:\pm\:$0.2081), with no comparable effect when µOR1 was coexpressed (ΔBRET_max_ = -0.2712$\:\pm\:$0.0059, LogΔBRET_50_ = -10.35$\:\pm\:$0.0636). However, the coexpression of CXCR4 did not influence S1P-induced Gα_i2_ activation (Fig. 2D). The inhibitory effect of S1P_1_ on CXCL12-induced Gα_i2_ activation extended to other Gα_i/_o subfamily members, including Gα_i1_, Gα_i3_, Gα_oA_, and Gα_oB_ (Additional file 1: Fig. S3). These findings demonstrate that S1P_1_ specifically interferes with CXCR4 activation without reciprocally affecting S1P_1_ activation. Furthermore, given that S1P_1_ coexpression did not alter CXCR4 surface expression, this modulation occurs through a functional mechanism rather than changes in receptor expression levels.

### S1P_1_ interferes with CXCR4-mediated Gα_i_ activation by sequestering Gα_i_ proteins

To investigate the effect of S1P_1_ stimulation on CXCR4-mediated signaling, we examined CXCL12-induced Gα_i2_ recruitment to CXCR4 in the presence or absence of S1P in HEK293A cells by utilizing a BRET-based biosensor, which measures an increase in the BRET ratio between Gα_i2_-Rluc8 and CXCR4-mCit [68]. CXCL12 increased Gα_i2_ recruitment to CXCR4 in cells expressing CXCR4 alone, but consistent with the aforementioned findings about reduced CXCL12-induced Gα_i2_ activation, CXCL12-induced Gα_i2_ recruitment to CXCR4 was significantly reduced in cells coexpressing CXCR4 and S1P_1_ (Fig. 2E). When cells expressing CXCR4 alone were cotreated with CXCL12 and S1P, the recruitment of Gα_i2_ to CXCR4 remained unaffected. Interestingly, in cells expressing both receptors, cotreatment with CXCL12 and S1P further reduced CXCL12-induced Gα_i2_ recruitment to CXCR4. We next investigated whether CXCR4 stimulation affects Gα_i2_ recruitment to S1P_1_ or not. S1P treatment induced Gα_i2_ recruitment to S1P_1_ in cells expressing S1P_1_ alone, and the coexpression of CXCR4 did not significantly influence S1P-induced Gα_i2_ recruitment to S1P_1_ (Fig. 2F). In cells expressing S1P_1_ alone, the addition of CXCL12 did not affect S1P-induced Gα_i2_ recruitment to S1P_1_. Even in cells expressing both CXCR4 and S1P_1_, Gα_i2_ recruitment to S1P_1_ remained unaffected by CXCL12 treatment. These results suggest that S1P, through S1P_1_, further interferes with CXCL12-induced Gα_i2_ recruitment to CXCR4, but CXCR4 has no discernible impact on S1P_1_ function under similar conditions.

To investigate whether S1P_1_ coexpression affects the basal association of Gα with CXCR4, we measured BRET between CXCR4-mCit and Gα_i2_-Rluc8 in the presence or absence of S1P_1_. Coexpression of S1P_1_ significantly reduced the basal association of Gα_i2_ with CXCR4 (Fig. 2G). In contrast, the expression of CXCR4 did not affect the basal association of Gα_i2_ with S1P_1_ (Fig. 2H). These results are consistent with prior observations, highlighting a unidirectional interference in which S1P_1_ interferes with CXCR4 function without reciprocal regulation.

Given that S1P_1_ stimulation further interferes with CXCR4, we next examined whether endogenous S1P contributes to this interference. To address this, we monitored CXCL12-induced Gα_i2_ activation in the presence of 10 µM of Ex26, a potent and selective S1P_1_ antagonist. In accordance with the results above, CXCL12-induced Gα_i2_ activation was significantly reduced in the presence of S1P_1_ when cells were pretreated with vehicle (Fig. 2I). Pretreatment with Ex26 did not affect CXCR4 activation in cells expressing CXCR4 alone. However, in cells coexpressing CXCR4 and S1P_1_, Ex26 partially restored CXCL12-induced Gα_i2_ activation, suggesting that endogenous S1P contributes to the interference of CXCR4 signaling by S1P_1_. Intriguingly, despite the partial restoration, CXCL12-induced Gα_i2_ activation was not fully recovered by Ex26 treatment. These observations suggest that the interference of CXCR4 signaling by S1P_1_ is not solely dependent on S1P_1_ activation by endogenous S1P but also involves the apo form of S1P_1_.

### S1P_1_ impairs CXCL12-induced CXCR4 oligomerization

**Fig. 3:**
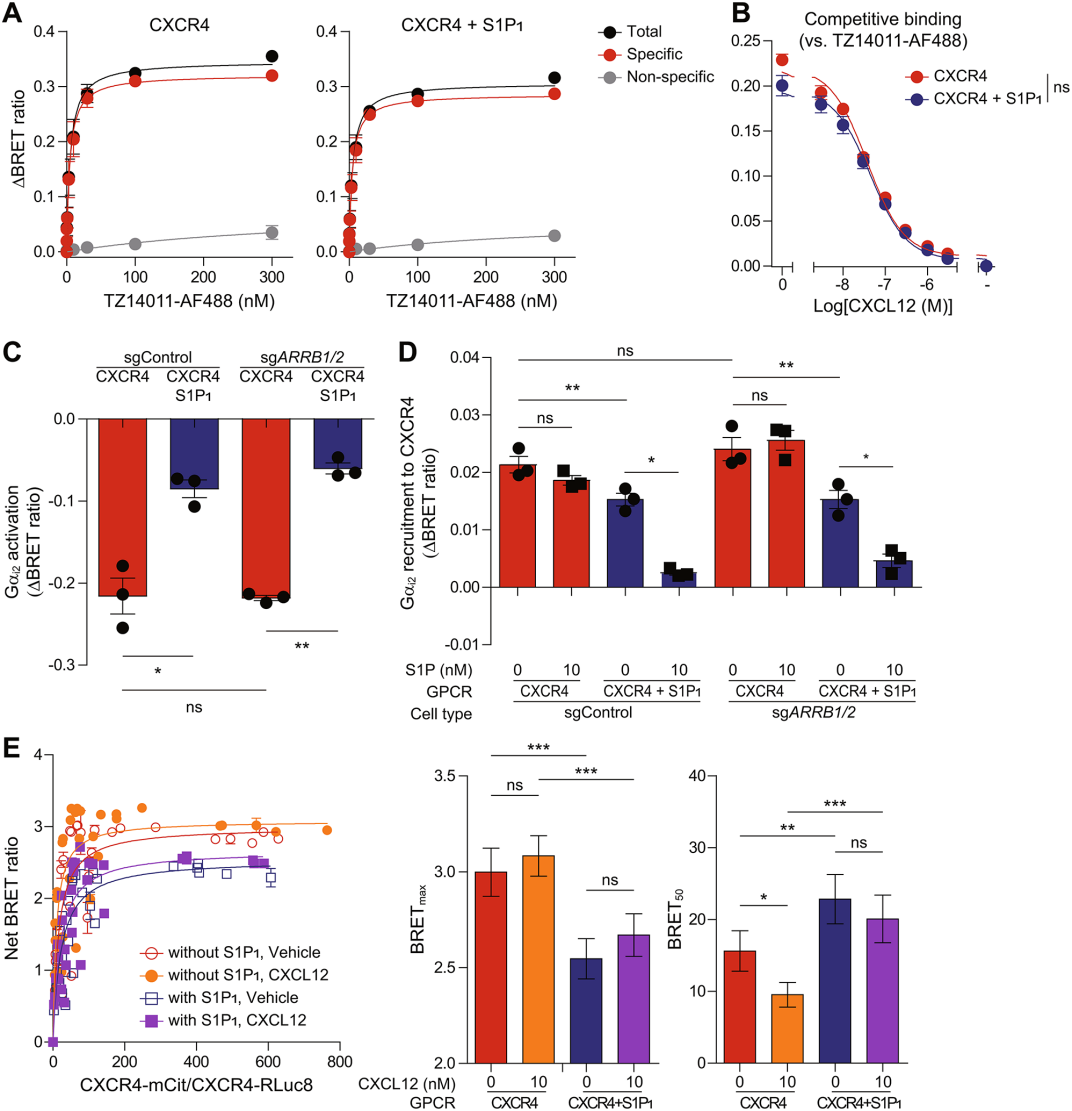
S1P_1_ impairs CXCR4 oligomerization without affecting ligand binding or β-arrestin-mediated desensitization. (**A**) BRET-based ligand binding assay was performed in HEK293A cells expressing Gluc-CXCR4 alone (left) or together with S1P_1_ (right). The selective CXCR4 antagonist TZ14011-AF488 was used as an acceptor to measure specific binding. IT1t (10 µM) was pretreated to account for non-specific BRET signal. Specific BRET signal was defined as the difference between the total BRET signal and the non-specific BRET signal. (**B**) Competitive binding was assessed using unlabeled CXCL12 (3 nM to 3 µM) in the presence of TZ14011-AF488 (10 nM) in cells expressing Gluc-CXCR4 alone or together with S1P_1_. (**C**,** D**) CXCL12 (10 nM)-induced Gα_i2_ activation (**C**) and Gα_i2_ recruitment to CXCR4 after cotreatment with S1P (10 nM) (**D**) were measured in control (sgControl) and β-arrestin1/2-deficient (sg*ARRB1/2*) HEK293A cells expressing CXCR4 alone or together with S1P_1_. (**E**) Quantitative BRET assay was performed in HEK293A cells between CXCR4-mCit and CXCR4-Rluc8 in the absence or presence of S1P_1_ and/or CXCL12 (10 nM). The curves were fitted using a non-linear regression equation, as a bar graph summarizing BRET_max_ and BRET_50_ of each condition. Data represent the mean ± SEM of *n* = 3 independent experiments (**A**-**D**) and the mean ± SD of *n* = 5 independent experiments (**E**). Statistical significance was tested using two-tailed Student’s *t*-test for *K*_D_ of specific binding (**A**) and for IC_50_ of the curves (**B**), two-way ANOVA followed by Bonferroni’s post-hoc test (**C**, **D**), and one-way ANOVA followed by Tukey’s post-hoc test (**E**). *P < 0.05; **P < 0.01; ***P < 0.001; ns, not significant

Since S1P_1_ continued to interfere with CXCR4 activation even when S1P_1_ stimulation was blocked, we further investigated the inhibitory mechanisms of S1P_1_ toward CXCR4. It has been reported that heteromerization of GPCRs can affect the ligand binding affinity of their partner GPCRs through allosteric modulation [69, 70]. To investigate the impact of heteromerization on the ligand binding affinity of CXCR4, we performed a BRET-based ligand binding assay by measuring the BRET ratio between Gaussia luciferase (Gluc)-tagged CXCR4 and the Alexa Fluor 488-conjugated CXCR4 antagonist TZ14011 (TZ14011-AF488) [21, 71]. To exclude the non-specific signal caused by random collisions between donor and acceptor, we pretreated cells with the high-affinity CXCR4 antagonist IT1t for 30 min. As expected, increasing the concentration of TZ14011-AF488 led to a linear increase in the BRET ratio in the presence of IT1t, but a hyperbolic increase in the BRET ratio in the absence of IT1t (Fig. 3A). The specific BRET signal, which was defined as the difference between the total BRET signal obtained in the absence of IT1t and the non-specific BRET signal obtained in the presence of IT1t, was saturated at nanomolar concentrations, indicating that TZ14011-AF488 interacts with Gluc-CXCR4 with high affinity. The equilibrium dissociation constant (*K*_D_) values between Gluc-CXCR4 and TZ14011-AF488 remained unaltered whether CXCR4 was expressed alone or coexpressed with S1P_1_ (*K*_D_ = 4.607 $\:\pm\:$ 0.8072 nM in cells expressing Gluc-CXCR4 alone; *K*_D_ = 4.507 $\:\pm\:$ 0.6392 nM in cells coexpressing Gluc-CXCR4 and S1P_1_). Subsequently, we performed a competitive ligand binding assay by treating cells with unlabeled CXCL12 to measure the dissociation of TZ14011-AF488 from Gluc-CXCR4. Similar concentrations of CXCL12 were required to dissociate TZ14011-AF488 from Gluc CXCR4, regardless of whether CXCR4 was expressed alone or coexpressed with S1P_1_ (IC_50_ = 38.30 $\:\pm\:$ 10.08 nM in cells expressing Gluc-CXCR4 alone; IC_50_ = 45.73 $\:\pm\:$ 13.19 nM in cells coexpressing Gluc-CXCR4 and S1P_1_) (Fig. 3B). These results suggest that the ligand binding affinity of CXCR4 is not affected by heteromerization with S1P_1_.

Traditionally, β-arrestins mediate GPCR desensitization by blocking G protein interactions and promoting receptor internalization [72]. However, some GPCRs can form super-complexes consisting of the GPCR, β-arrestin, and G protein, enabling sustained signaling within intracellular compartments [11]. Notably, heteromerization with other GPCRs has been shown to enhance β-arrestin recruitment, leading to the inhibition of the primary receptor’s function [17, 21, 73, 74]. In light of these findings, we examined the effect of S1P_1_ on β-arrestin recruitment to CXCR4. Increasing the amounts of mCit-β-arrestin2 with a fixed amount of CXCR4-Rluc8 resulted in hyperbolic increases in the BRET ratio (Additional file 1: Fig. S4A), indicating complex formation between CXCR4 and β-arrestin2. Coexpression of S1P_1_ slightly but significantly increased BRET_max_ values for the CXCR4-β-arrestin2 complex (BRET_max_ = 0.2138 ± 0.0128 in cells expressing CXCR4-Rluc8 alone; BRET_max_ = 0.2771 ± 0.0085 in cells coexpressing CXCR4-Rluc8 and Myc-S1P_1_), suggesting that the amount of the CXCR4-β-arrestin2 complex increases in the presence of S1P_1_. Furthermore, S1P treatment enhanced β-arrestin2 recruitment to CXCR4 in the presence of S1P_1_ (Additional file 1: Fig. S4B), whereas CXCL12 treatment did not affect β-arrestin2 recruitment to S1P_1_ in the presence of CXCR4 (Additional file 1: Fig. S4C). These results suggest that S1P-induced β-arrestin2 recruitment to CXCR4 may contribute to the interference of CXCR4 signaling by the S1P/S1P_1_ axis. However, CXCR4-mediated G protein activation and G protein recruitment to CXCR4 were still inhibited by the S1P/S1P_1_ axis in β-arrestin1/2-deficient HEK293A cells, similar to what was observed in control cells (Fig. 3C, D, Additional file 1: Fig. S4D). These observations suggest the possibility of an alternative inhibitory mechanism by S1P_1_ toward CXCR4 that operates independently of β-arrestin involvement. Notably, CXCR4-mediated G protein activation and G protein recruitment to CXCR4 were not increased in β-arrestin1/2-deficient cells (Fig. 3C, D), indicating that CXCR4 desensitization and internalization are largely independent of β-arrestins. This conclusion is supported by recent findings showing that CXCL12-induced CXCR4 internalization remains unaffected in β-arrestin1/2-deficient cells [75].

CXCR4 is known to form dimers and oligomers, and perturbations in CXCR4 oligomerization have been shown to disrupt its signaling and function [18, 23, 34, 35]. Interestingly, coexpression with CD4 diminishes CXCL12-induced CXCR4 nanoclustering, leading to impaired CXCR4-mediated signaling and migration in Jurkat cells [34]. To investigate the effect of S1P_1_ on CXCR4 oligomerization, we conducted a quantitative BRET assay. The coexpression of S1P_1_ increased the BRET_50_ and decreased the BRET_max_ values for CXCR4-Rluc8 and CXCR4-mCit (Fig. 3E; Table 1), indicating reduced affinity between CXCR4 protomers and decreased oligomerization at steady state. Notably, CXCL12 treatment lowered the BRET_50_ for CXCR4 homomers only in the absence of S1P_1_, suggesting that S1P_1_ interferes with the CXCL12-induced increase in affinity between CXCR4 protomers. These findings suggest that S1P_1_ affects CXCR4 function by disrupting its oligomerization.

**Table 1:** BRET_max_ and BRET_50_ values from the quantitative BRET assay between CXCR4-Rluc8 and CXCR4-mCit

	Without S1P_1_		With S1P_1_	
	Vehicle	CXCL12	Vehicle	CXCL12
BRET_max_	2.998 ± 0.1255	3.083 ± 0.1056	2.546 ± 0.1049	2.670 ± 0.1109
BRET_50_	15.61 ± 2.814	9.534 ± 1.716	22.84 ± 3.430	20.07 ± 3.315

### TM3 of S1P_1_ is important for the modulation of CXCR4 within the heteromer complex

**Fig. 4:**
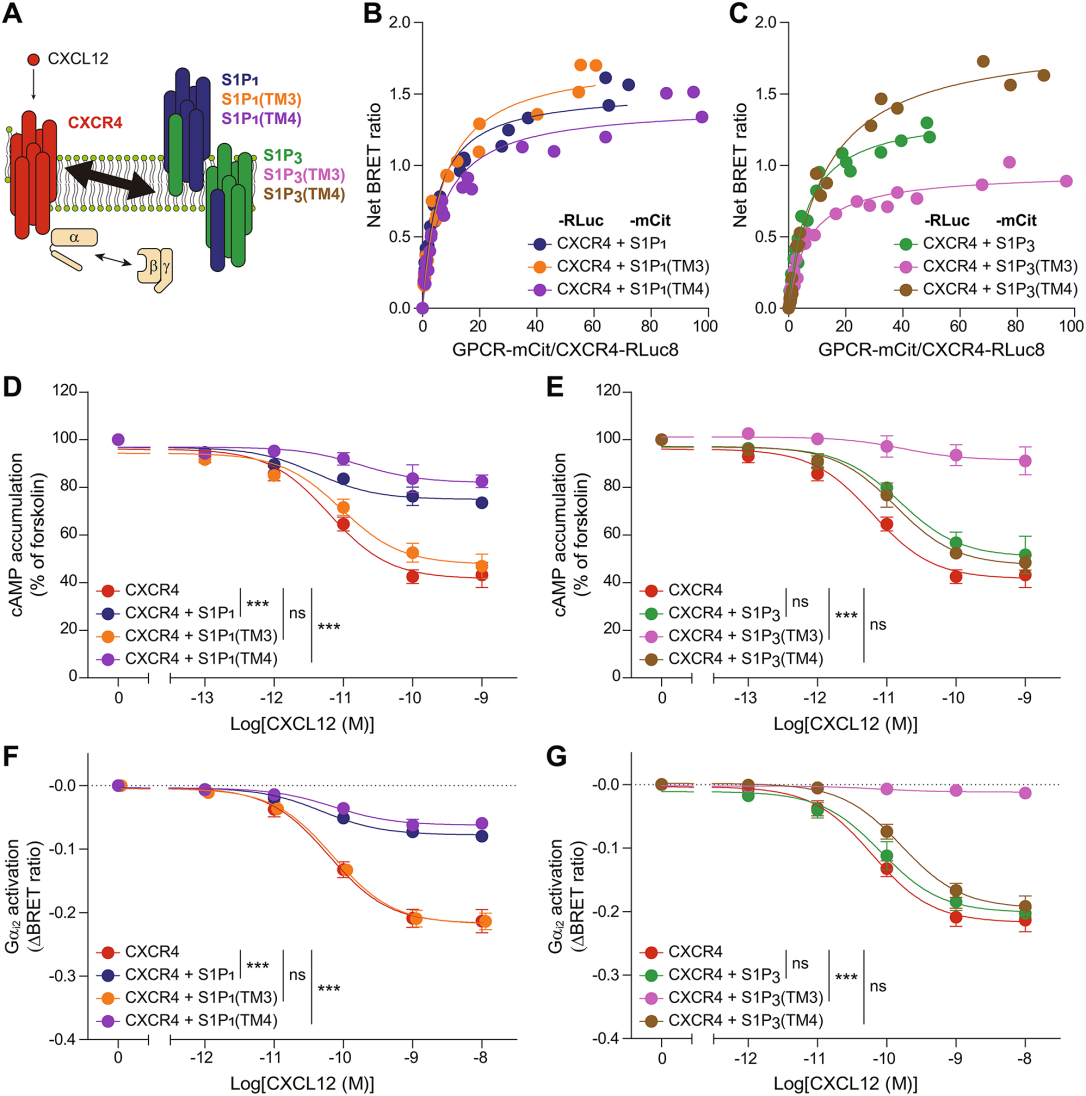
Identification of the S1P_1_ domain responsible for CXCR4 inhibition by S1P_1_. (**A**) Schematic diagram of CXCL12-induced G protein activation in HEK293A cells coexpressing CXCR4 and wildtype or domain-swapped mutants of S1P_1_ or S1P_3_. (**B**,** C**) Quantitative BRET assay was performed between CXCR4-Rluc8 and mCit-tagged S1P_1_, S1P_1_(TM3), or S1P_1_(TM4) (**B**) and mCit-tagged S1P_3_, S1P_3_(TM3), or S1P_3_(TM4) (**C**). The curves were fitted using a non-linear regression equation and represent three independent experiments. (**D**,** E**) CXCL12-induced cAMP signaling was measured in HEK293A cells expressing CXCR4 alone or together with S1P_1_, S1P_1_(TM3), or S1P_1_(TM4) (**D**) or together with S1P_3_, S1P_3_(TM3), or S1P_3_(TM4) (**E**). (**F**,** G**) CXCL12-induced Gα_i2_ activation was measured in HEK293A cells expressing CXCR4 alone or together with S1P_1_, S1P_1_(TM3), or S1P_1_(TM4) (**F**) or together with S1P_3_, S1P_3_(TM3), or S1P_3_(TM4) (**G**). Data represent the mean ± SEM of *n* = 4 independent experiments. Statistical significance was tested using two-way ANOVA followed by Bonferroni’s post-hoc test (**D**-**G**). ***P < 0.001; ns, not significant

Previous studies have demonstrated the significance of TM4 of S1P_1_ in its interaction with CD69 and crosstalk with LPA_1_ [17, 52]. For dopamine receptor D2 (D2R), TM4 and TM5 form the interaction interface of the D2R dimer [76, 77], and TM3 is also crucial for dimer formation [78]. To investigate the interaction interface between CXCR4 and S1P_1_, we generated domain-swapped mutants of S1P_1_. Because S1P_3_ showed BiFC signals with CXCR4 (Fig. 1C) and is reported to promote CXCR4 activation [79], we constructed S1P_1_(TM3) and S1P_1_(TM4) mutants by replacing TM3 and TM4 of S1P_1_ with the corresponding domains from S1P_3_ (Fig. 4A). We also generated S1P_3_(TM3) and S1P_3_(TM4) mutants by replacing TM3 and TM4 of S1P_3_ with the corresponding domains from S1P_1_. Immunocytochemistry and flow cytometry analysis confirmed that MYC-tagged S1P_1_, S1P_3_, and their domain-swapped mutants were properly expressed on the surface of HEK293A cells (Additional file 1: Fig. S5A, B). Subsequently, we investigated whether CXCR4 forms heteromers with S1P_1_, S1P_3_, or the domain-swapped mutants through quantitative BRET analysis. Increasing the amounts of mCit-tagged S1P_1_, S1P_3_, or the mutants with a fixed amount of CXCR4-Rluc8 all led to hyperbolic increases in the BRET ratio (Fig. 4B, C; Table 2). These results suggest that CXCR4 indeed forms heteromers with S1P_1_ and S1P_3_, regardless of whether TM3 or TM4 is replaced with the corresponding domain from S1P_3_ and S1P_1_, respectively.

**Table 2:** BRET_max_ and BRET_50_ values from the quantitative BRET assay between CXCR4-Rluc8 and mcit-tagged S1P_1_, S1P_3_, or the domain-swapped mutants

-Rluc8	CXCR4					
-mCit	S1P_1_	S1P_1_(TM3)	S1P_1_(TM4)	S1P_3_	S1P_3_(TM3)	S1P_3_(TM4)
BRET_max_	1.523 ± 0.0284	1.745 ± 0.0379	1.422 ± 0.0314	1.373 ± 0.0253	0.955 ± 0.0175	1.903 ± 0.0327
BRET_50_	5.169 ± 0.3383	7.237 ± 0.5014	7.191 ± 0.5593	6.471 ± 0.3773	6.968 ± 0.4726	12.69 ± 0.6873

We next examined the influence of the domain-swapped mutants of S1P_1_ and S1P_3_ on CXCR4 activation by analyzing CXCL12-induced cAMP signaling and Gα_i2_ activation in HEK293A cells coexpressing CXCR4 with either S1P_1_, S1P_3_, or each of the mutants. As previously mentioned, CXCL12-induced cAMP signaling was notably reduced when S1P_1_ was coexpressed (Fig. 4D). Interestingly, the coexpression of S1P_1_(TM3) did not significantly affect CXCL12-induced cAMP signaling, while S1P_1_(TM4) coexpression had a similar effect on CXCL12-induced cAMP signaling as S1P_1_ coexpression. These observations suggest that TM3 of S1P_1_ is necessary for CXCR4 regulation by S1P_1_, while TM4 of S1P_1_ is not. Although S1P_3_ forms heteromers with CXCR4, this interaction did not significantly affect CXCL12-induced cAMP signaling (Fig. 4E). Additionally, we observed that CXCL12-induced cAMP signaling was significantly reduced when S1P_3_(TM3) was coexpressed, but not when S1P_3_(TM4) was coexpressed, highlighting the importance of TM3 of S1P_1_ in CXCR4 inhibition by S1P_1_. Similarly, CXCL12-induced Gα_i2_ activation was significantly reduced when S1P_1_, S1P_1_(TM4), or S1P_3_(TM3) were coexpressed, but not when S1P_1_(TM3), S1P_3_, or S1P_3_(TM4) were coexpressed (Fig. 4F, G). The inhibition of CXCR4 by S1P_1_ was not due to a decrease in CXCR4 expression, as flow cytometry analysis revealed no significant alteration in the surface expression of CXCR4 in HEK293A cells expressing CXCR4 alone or in combination with S1P_1_, S1P_3_, or any of the mutants (Additional file 1: Fig. S5C). Taken together, these findings suggest that TM3 of S1P_1_ is the regulatory motif for CXCR4 within the heteromer complex.

### S1P_1_ coexpression interferes with CXCR4-mediated intracellular signaling and cell migration in Jurkat cells

**Fig. 5:**
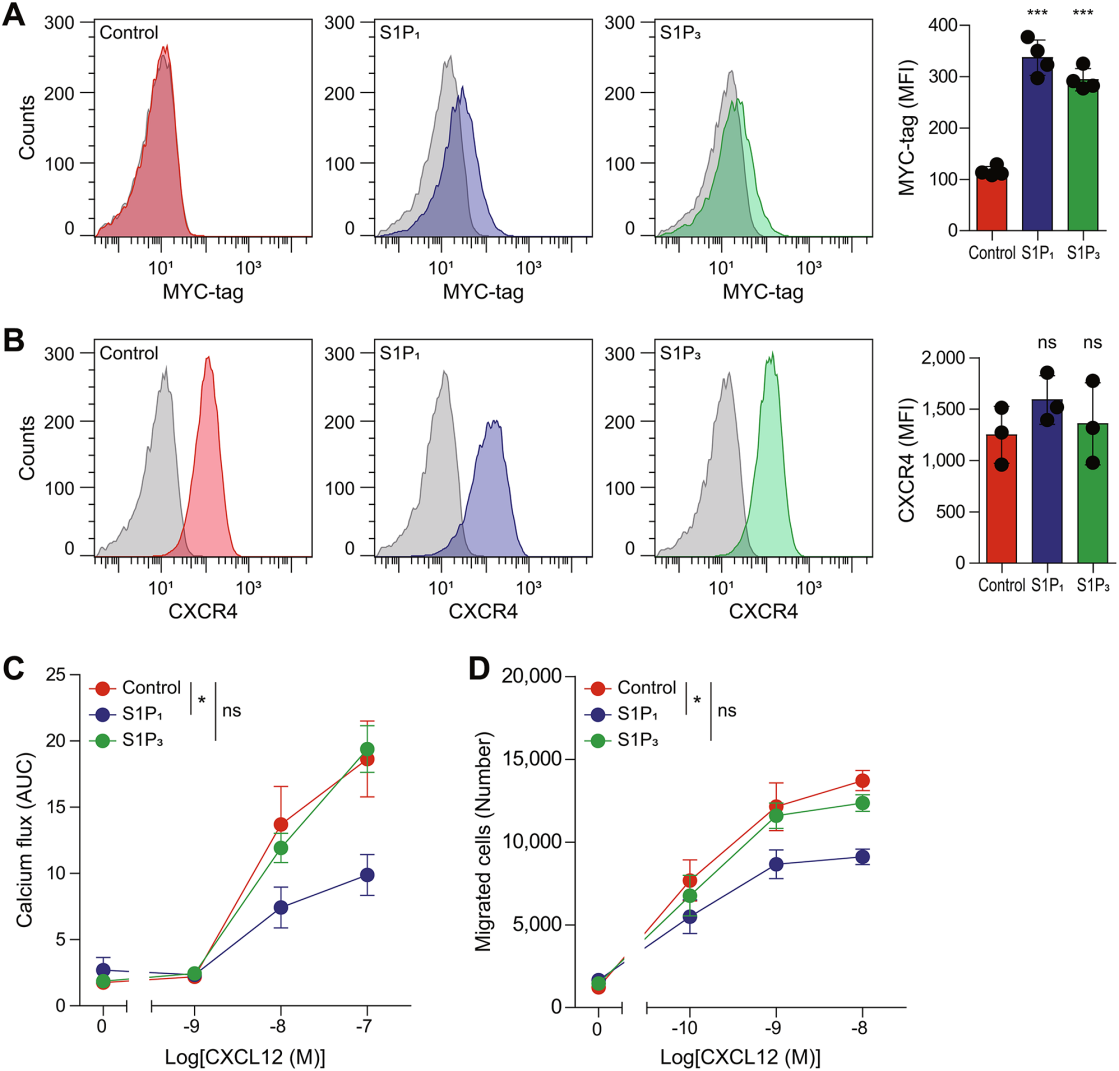
Inhibition of CXCR4-mediated signaling and migration by S1P_1_ in Jurkat cells. (**A**,** B**) Surface expression of S1P_1_, S1P_3_, and CXCR4 in Jurkat cells overexpressing Myc-S1P_1_ or Myc-S1P_3_. Anti-MYC-tag (9B11) antibody was used to detect S1P_1_ and S1P_3_ (**A**) and anti-CXCR4 (Ulocuplumab) antibody was used to detect CXCR4 (**B**) in the control (red), S1P_1_ (blue), and S1P_3_ (green) overexpressing cells. Gray histograms represent staining with IgG control antibody. Representative histograms of four independent experiments are shown. Data represent the mean ± SD of *n* = 4 independent experiments. (**C**,** D**) CXCL12-induced intracellular calcium flux (**C**) and cell migration (**D**) were detected in the control (red), S1P_1_ (blue), and S1P_3_ (green) overexpressing cells after treatment with CXCL12 in a dose-dependent manner. Data represent the mean ± SEM of *n* = 3 to 4 independent experiments. Statistical significance was tested using one-way ANOVA followed by Dunnett’s post-hoc test (**A**, **B**) and two-way ANOVA followed by Bonferroni’s post-hoc test (**C**, **D**). *P < 0.05; ***P < 0.001; ns, not significant

To further investigate the influence of S1P_1_ on CXCR4, we established an S1P_1_-overexpressing cell line using lentivirus encoding MYC-tagged S1P_1_ in Jurkat T lymphoblast cells, which are widely studied in CXCR4-mediated cell migration and signaling [80, 81]. Flow cytometry analysis confirmed the increased surface expression of MYC-tagged S1P_1_ in the S1P_1_-expressing (hereafter “S1P_1_”) Jurkat cells compared to the control cells, which were transduced with lentivirus encoding empty vector (Fig. 5A). The surface expression of CXCR4 did not change significantly in cells overexpressing S1P_1_ (Fig. 5B). Interestingly, intracellular calcium flux and cell migration toward CXCL12 were significantly impaired in the S1P_1_ Jurkat cells compared to the control cells (Fig. 5C, D). To validate the role of S1P_1_ in interfering with CXCR4-mediated cell migration, we stably overexpressed S1P_3_ in Jurkat cells as a negative control. Flow cytometry analysis confirmed the proper surface expression of MYC-tagged S1P_3_ in the S1P_3_-expressing (hereafter “S1P_3_”) Jurkat cells (Fig. 5A). The surface expression of CXCR4 remained consistent between the control and S1P_3_ Jurkat cells (Fig. 5B). In contrast to the findings observed in the S1P_1_ Jurkat cells, CXCL12-induced intracellular calcium flux and cell migration were not altered significantly in the S1P_3_ Jurkat cells compared to the control cells (Fig. 5C, D). Consistent with the results in G protein activation in HEK293A cells (Fig. 4D-F), these results indicate that S1P_1_ specifically interferes with CXCR4 activity in Jurkat cells, while S1P_3_ does not exhibit an inhibitory effect.

### S1P_1_ forms heteromers with CXCR4 and interferes with cell migration toward CXCL12 in T cells endogenously expressing both receptors

KARPAS299 is a human non-Hodgkin’s lymphoma T cell line that inherently expresses both CXCR4 and S1P_1_ [67, 82]. Previous studies have proposed neutralizing CXCR4 as a therapeutic approach for non-Hodgkin’s lymphoma [83, 84] and have elucidated CXCR4-mediated cell migration in KARPAS299 cells [85]. Given the endogenous coexpression of CXCR4 and S1P_1_ in these cells, we investigated the heteromerization of these GPCRs and the effect of S1P_1_ on CXCR4-mediated functions. To examine whether S1P_1_ directly interferes with CXCR4 activity, we deleted the *S1PR1* gene in KARPAS299 cells using the CRISPR/Cas9 system.

**Fig. 6:**
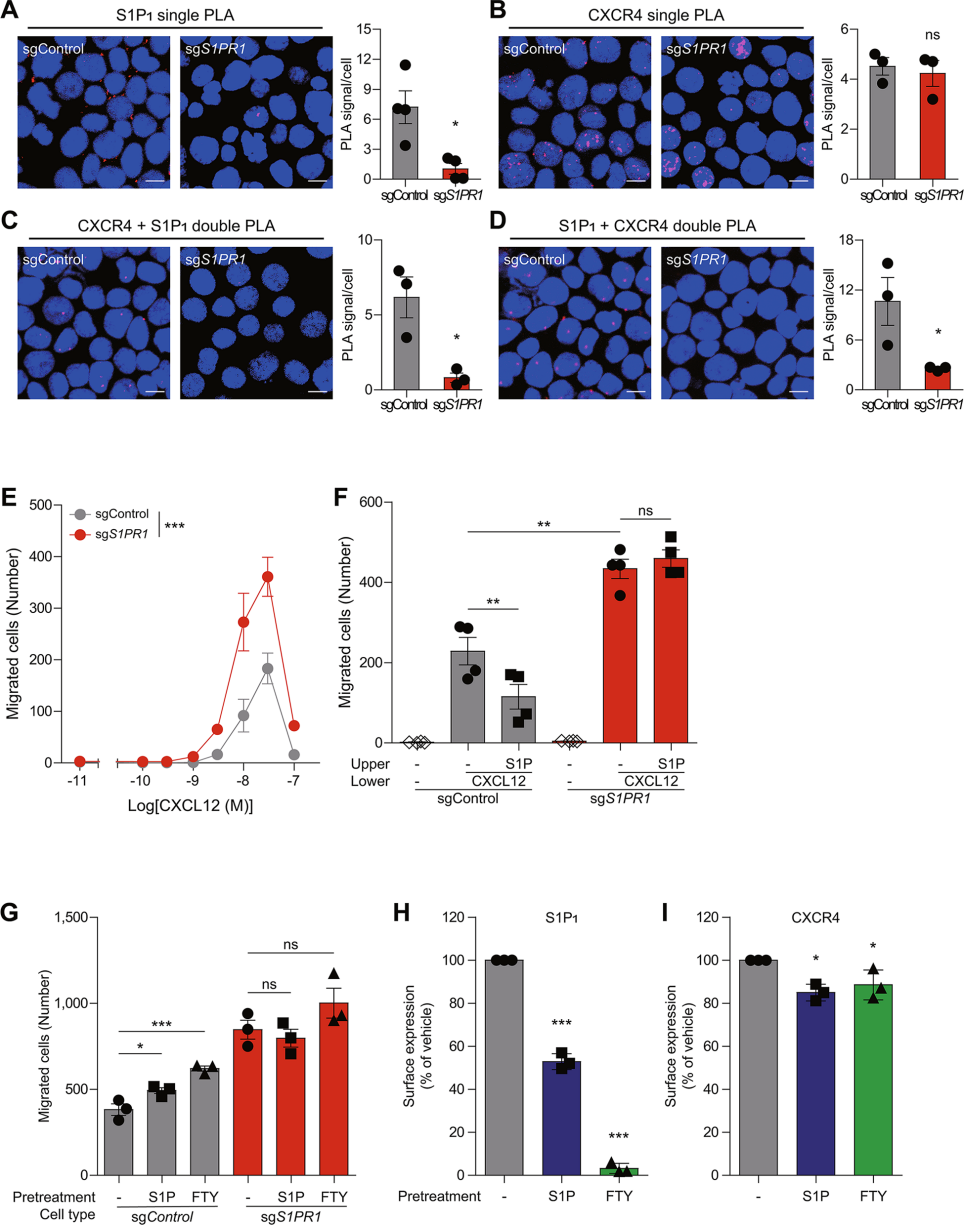
S1P/S1P_1_ axis inhibits CXCR4-mediated migration of KARPAS299 cells endogenously expressing CXCR4 and S1P_1_ through heteromer formation. (**A-D**) Detection of CXCR4-S1P_1_ heteromers in control and S1P_1_-deficient KARPAS299 cells. KARPAS299 cells were transduced with lentivirus encoding CRISPR/Cas9 and sgControl or sg*S1PR1*, and cells were stained with anti-S1P_1_ (#218713) antibody alone for both probes 1 and 2 (**A**), anti-CXCR4 (Ulocuplumab) antibody alone for both probes 1 and 2 (**B**), anti-CXCR4 for probe 1 and anti-S1P_1_ for probe 2 (**C**), and anti-S1P_1_ for probe 1 and anti-CXCR4 for probe 2 (**D**) to identify S1P_1_ homomers, CXCR4 homomers, and S1P_1_-CXCR4 heteromers. Images are representative of three independent experiments. Scale bars: 10 μm. (**E**,** F**) Transwell migration assays were performed in control and S1P_1_-deficient KARPAS299 cells. The effect of S1P_1_ on CXCL12-induced migration (**E**) and the effect of S1P (10 nM) on CXCL12 (30 nM)-induced migration (**F**). (**G-I**) The effect of S1P_1_ agonist, S1P (10 nM) and FTY720P (100 nM), pretreatment (3 h) on CXCL12-induced migration and the surface expression of S1P_1_ and CXCR4 were assessed in KARPAS299 cells. Transwell migration assays were performed in control and S1P_1_-deficient KARPAS299 cells (**G**) and flow cytometry analysis was conducted to analyze surface expression of S1P_1_ (**H**) and CXCR4 (**I**) in KARPAS299 cells using anti-S1P_1_ (#218713) and anti-CXCR4 (Ulocuplumab) antibodies. Data represent the mean ± SEM of *n* = 3 to 4 independent experiments (**A**-**G**) and the mean ± SD of *n* = 3 independent experiments (**H**, **I**). Statistical significance was tested using two-tailed Student’s *t*-test (**A**-**D**), two-way ANOVA followed by Bonferroni’s post-hoc test (**E**, **F**), and one-way ANOVA followed by Dunnett’s post-hoc test with vehicle-treated condition (**G**-**I**). *P < 0.05; **P < 0.01; ***P < 0.001; ns, not significant

Single PLA and flow cytometry revealed a significant reduction in S1P_1_ expression in sg*S1PR1*-treated cells (referred to as “S1P_1_-deficient cells”) compared to sgControl-treated cells (referred to as “control cells”) (Fig. 6A, Additional file 1: Fig. S6A), while CXCR4 expression remained unaffected (Fig. 6B, Additional file 1: Fig. S6B). Additionally, S1P-induced migration was markedly diminished in S1P_1_-deficient cells, confirming the effective downregulation of S1P_1_ (Additional file 1: Fig. S6C). CXCR4-S1P_1_ double PLA signals were observed in control cells, but were absent in S1P_1_-deficient cells, supporting the presence of CXCR4-S1P_1_ heteromers in KARPAS299 cells (Fig. 6C, D).

Interestingly, CXCL12-induced migration was significantly increased in S1P_1_-deficient cells (Fig. 6E, F). Since CXCR4 expression levels remained unchanged, this increase likely results from the loss of S1P1’s regulatory effect on CXCR4. Consistent with our observation that S1P_1_ stimulation further inhibited CXCR4 function in HEK293A cells (Fig. 2E), the addition of S1P to the upper chamber reduced KARPAS299 cell migration toward CXCL12 in the lower chamber in control cells, but not in S1P_1_-deficient cells (Fig. 6F). This finding indicates that S1P interferes with CXCL12-induced migration through S1P_1_. In contrast, adding CXCL12 to the upper chamber did not affect S1P-induced migration in KARPAS299 cells (Additional file 1: Fig. S6D), consistent with the unidirectional inhibition of CXCR4 function by S1P_1_ observed in HEK293A cells. Additionally, Ex26, at concentration that completely blocks S1P-induced migration, did not alter the CXCL12-induced migration toward the lower chamber (Additional file 1: Fig. S6D, E). This suggests that Ex26 may be insufficient to induce conformational changes in S1P_1_ that could impact CXCR4-S1P_1_ heteromers. Collectively, these results demonstrate that the S1P/S1P_1_ axis attenuates CXCR4 function through the formation of CXCR4-S1P_1_ heteromers in KARPAS299 cells.

A previous study showed that FTY720, an agonist and functional inhibitor of S1P_1_, enhanced CXCR4-dependent migration, calcium mobilization, and actin-polymerization in hematopoietic cells in vitro when cells were treated with FTY720 for 3 h [47]. To evaluate the effect of FTY720 in KARPAS299 cells, cells were pretreated with or without S1P or FTY720P, the active form of FTY720, for 3 h, and CXCR4-mediated migration was assessed using a transwell migration assay. Both S1P and FTY720P significantly increased CXCL12-induced migration in control cells, while this enhancement was absent in S1P_1_-deficient cells (Fig. 6G), suggesting that the effect depends on S1P_1_. Since S1P_1_ agonists are known to induce S1P_1_ internalization, the surface expression of S1P_1_ was analyzed after 3 h of pretreatment with these agonists. Both S1P and FTY720P substantially decreased surface S1P_1_ expression (Fig. 6H). Although CXCR4 surface expression was also slightly reduced, possibly due to the co-internalization of CXCR4-S1P_1_ heteromers (Fig. 6I), CXCL12-induced migration was enhanced, resembling the increase observed in S1P_1_-deficient cells. In summary, these findings indicate that while S1P/S1P_1_ axis interferes with CXCR4 function through heteromerization, prolonged treatment of S1P_1_ agonists restores CXCR4 function by internalizing S1P_1_ from the cell surface.

**Fig. 7:**
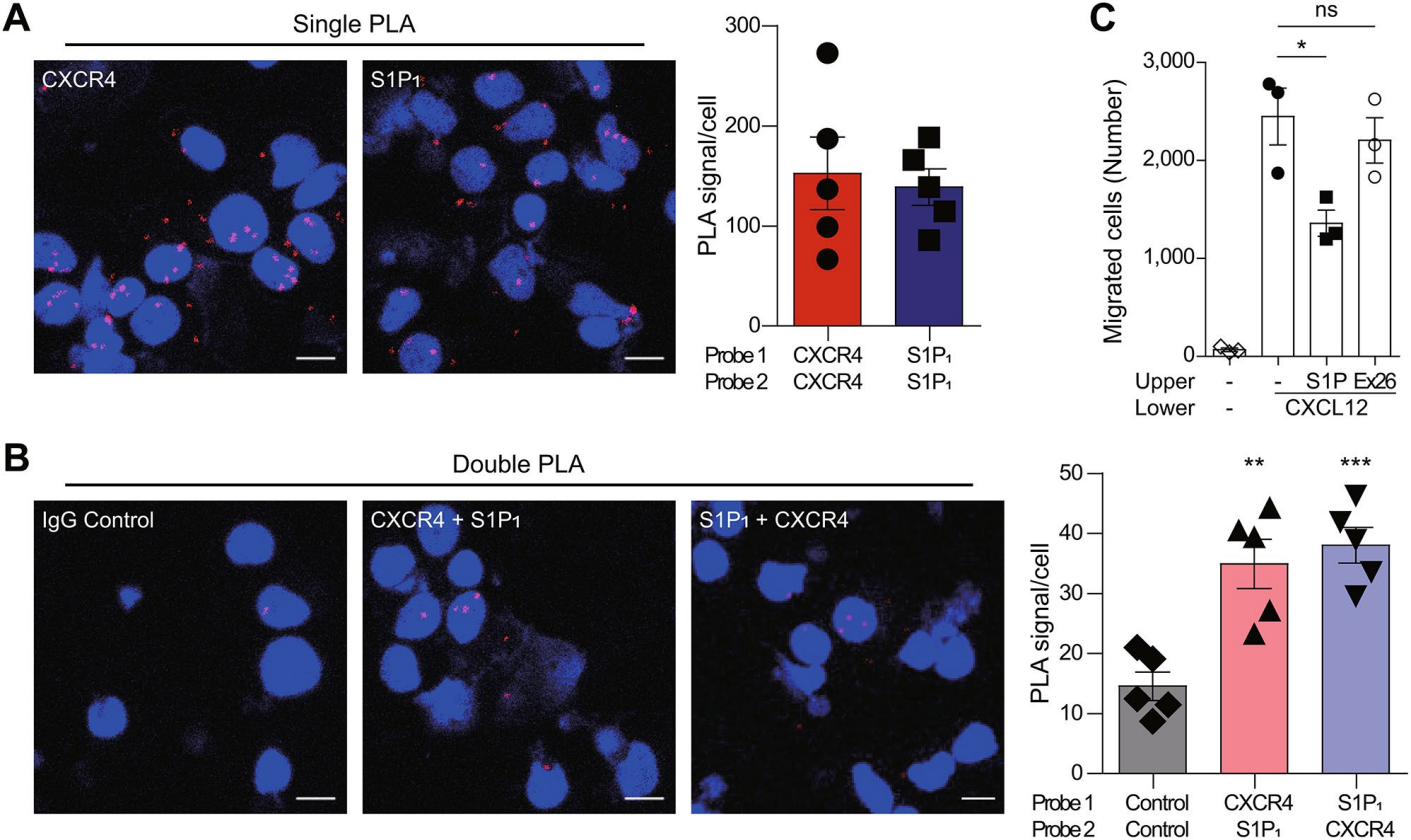
Presence of CXCR4-S1P_1_ heteromers and inhibition of CXCR4-mediated migration by S1P/S1P_1_ axis in primary T cells. (**A**) Detection of CXCR4 and S1P_1_ homomers in primary T cells. Cells were stained with anti-CXCR4 (Ulocuplumab) or anti-S1P_1_ (#218713) antibody alone for probe 1 and 2. (**B**) Detection of CXCR4-S1P_1_ heteromers in primary T cells. Cells were stained with human IgG control antibody for probe 1 and 2 (left), anti-CXCR4 for probe 1 and anti-S1P_1_ for probe 2 (middle), and anti-S1P_1_ for probe 1 and anti-CXCR4 for probe 2 (right). Images are representative of three independent experiments. Scale bars: 10 μm. (**C**) The effect of S1P (10 nM) and S1P_1_ antagonist Ex26 (10 µM) on CXCL12 (30 nM)-induced migration was measured in primary T cells. Data represent the mean ± SEM of *n* = 3 to 5 independent experiments. Statistical significance was tested using two-tailed Student’s *t*-test (**A**) and one-way ANOVA followed by Dunnett’s post-hoc test (**B**, **C**). *P < 0.05; **P < 0.01; ***P < 0.001; ns, not significant

To further examine the impact of S1P_1_ on CXCR4-mediated retention signaling and cell migration in T lymphocytes, we isolated primary T cells from peripheral blood mononuclear cells (PBMCs). Flow cytometry analysis confirmed the isolation of CD4+ T cells from PBMCs (Additional file 1: Fig. S7A, B) and revealed uniform expression of CXCR4 in primary T cells (Additional file 1: Fig. S7C). However, the majority of primary T cells did not express S1P_1_, with a small number of cells expressing high and low levels of S1P_1_ (Additional file 1: Fig. S7D), reflecting S1P_1_ internalization due to high levels of S1P in peripheral blood [86]. Single PLA signals for CXCR4 and S1P_1_ were observed in primary T cells at comparable levels (Fig. 7A), confirming the expression of both receptors in these cells. Double PLA signals were also observed, suggesting the presence of CXCR4-S1P_1_ heteromers (Fig. 7B). Consistent with the results of CXCR4-mediated cell migration in KARPAS299 cells, stimulation of cells in the upper chamber with S1P interfered with cell migration toward CXCL12, but not with Ex26 (Fig. 7C). The modulation of CXCR4 by S1P_1_ stimulation was less pronounced in primary T cells compared to KARPAS299 cells, probably due to the lack of S1P_1_ expression in most T cells. Taken together, these results suggest that S1P_1_ forms heteromers with CXCR4 and interferes with CXCR4-mediated cell migration also in primary T cells.

## Discussion

In this study, we identified and validated the heteromerization between CXCR4 and S1P_1_ using BiFC, BRET, and PLAs in HEK293A, KARPAS299, and primary T cells. Our findings demonstrated that the S1P/S1P_1_ axis interferes with CXCR4-mediated Gα_i_ activation, cAMP responses, calcium signaling, and migration without altering CXCR4 surface expression or its binding to CXCL12. Conversely, CXCL12/CXCR4 did not affect S1P_1_-mediated functions. This modulatory effect of S1P_1_ on CXCR4 is mediated by sequestering G proteins away from CXCR4 and reducing CXCR4 oligomerization, with S1P_1_’s TM3 domain being essential for this regulation. Furthermore, pretreatment with FTY720P enhanced CXCL12-induced migration by reducing the surface expression of S1P_1_.

Using quantitative BRET, we observed reduced affinity between CXCR4 protomers and decreased oligomerization in the presence of S1P_1_ (Fig. 3E). CXCR4 exists as a mixture of monomers, dimers and oligomers, with its homomerization closely linked to signaling activity. Studies have demonstrated that increased CXCR4 expression enhances oligomerization and basal activity in immune and cancer cells [18, 34, 35]. Additionally, CXCR4 interacts with other GPCRs and cell surface molecules, including CCR2, CCR5, CXCR7, and CD4, and its function is regulated by these heteromerization [36]. CXCL12 stimulation further promotes CXCR4 dimerization and oligomerization, whereas perturbations such as pertussis toxin treatment or TM6 mutations attenuate CXCR4 activity and oligomerization [18, 23, 34]. Notably, CXCR4 homomerization occurs prior to G protein activation in response to CXCL12 stimulation [87]. Importantly, disruption of CXCR4 oligomers has been shown to impair cell migration and pro-survival signaling while sensitizing malignant cells to therapeutic agents such as venetoclax, a Bcl-2 inhibitor [88]. These findings collectively suggest that CXCR4 oligomers are more effective in mediating signaling than monomers and reinforce the conclusion that S1P_1_ negatively regulates CXCR4 signaling and function by inhibiting CXCR4 oligomerization.

β-Arrestins play a key role in GPCR desensitization and compete with G proteins for GPCR binding [89, 90]. Several studies have reported β-arrestin cross-recruitment regulated by GPCR heteromerization. For example, angiotensin type 1 A receptor activation enhances β-arrestin interaction with the β2AR [74], and LPA_1_ activation induces β-arrestin recruitment to S1P_1_, thereby impairing S1P_1_/Gα_i_ signaling in endothelial junctions [17]. In our study, we observed increased interaction between CXCR4 and β-arrestins, as well as β-arrestin cross-recruitment to CXCR4 in the presence of S1P_1_ (see Additional file 1: Fig. S4A, B). Despite these interactions, CXCR4-mediated Gα_i2_ activation and recruitment were still interfered by the S1P/S1P_1_ axis, even in β-arrestin1/2-deficient HEK293A cells (see Fig. 3C, D). These findings suggest that S1P_1_ directly modulates CXCR4 function through heteromer formation. CXCR4 has been shown to form high-order oligomers, or nanoclusters, which are essential for its full activation in T cells and cancer cells [18, 34]. Our results demonstrated that S1P_1_ decreases CXCR4-Gα_i_ pre-association and reduces CXCR4 oligomerization by reducing the affinity between CXCR4 protomers (see Figs. 2G and 3E). Given that previous study suggests CXCR4 homomerization occurs prior to G protein activation [87], our findings indicate that S1P_1_ may reduce CXCR4-Gα_i_ pre-association by altering CXCR4 oligomerization.

To define the interaction interface between CXCR4 and S1P_1_, we initially employed synthetic peptides designed to mimic the TM sequences of the two GPCRs [91], but these peptides were ineffective under our experimental conditions. Through the construction of domain-swapped mutants, wherein TM3 or TM4 of S1P_1_ was replaced with the corresponding TM of S1P_3_, we identified TM3 of S1P_1_ as responsible for the modulation of CXCR4 within the heteromer complex. Since S1P_1_ forms heteromers with CXCR4 and interferes with CXCR4-mediated Gα_i_ activation without affecting CXCL12 binding to CXCR4, it is plausible that TM3 of S1P_1_ plays a crucial role in disrupting CXCR4 oligomerization and G protein activation. Further studies should focus on elucidating the detailed molecular mechanisms and identifying specific domains or residues responsible for the modulation of CXCR4 by S1P_1_.

Previous studies have shown that β2ARs expressed on lymphocytes enhance CXCR4-mediated retention signals by forming heteromers, thereby promoting lymphocyte retention in LNs [16, 28]. In contrast, other studies have demonstrated that S1P_1_ counteracts CXCR4-mediated retention signals, facilitating the egress of HSPCs from the BM toward the S1P gradient [45, 46, 92, 93]. In this context, immune cell egress from lymphoid organs is influenced by the balance between the retention signal mediated by CXCL12/CXCR4 and the egress-promoting signal driven by S1P/S1P_1_. Our study identifies a direct mechanism of heteromer-based modulation of CXCR4 activation by S1P/S1P_1_ in both KARPAS299 T cell lymphoma and primary T cells. These findings unveil an unexplored role for S1P_1_, wherein it not only promotes immune cell migration along the S1P gradient in peripheral blood but also actively disrupts the CXCR4-mediated retention signal through heteromer formation.

S1P_1_ has been implicated in various health conditions, including autoimmune diseases, inflammatory diseases, neurodegenerative disorders, and cancers such as breast cancer, pancreatic cancer, and glioblastoma [94, 95]. This has spurred the development of several S1P_1_-targeted ligands for therapeutic use. For example, FTY720, a clinically approved prodrug of FTY720P for multiple sclerosis, has shown efficacy in various cancer models [96]. Additionally, FTY720 exhibits neuroprotective effects in mouse models of Parkinson’s disease, while S1P_1_ agonist SEW2871 treatment improves cognitive function in rat models of Alzheimer’s disease [97, 98]. Recent studies suggest a complex interplay between CXCR4 and S1P_1_ signaling in conditions such as multiple myeloma, glioblastoma, and neurodegenerative diseases, where CXCR4 is a primary therapeutic target [99–101]. Notably, mice deficient in S1P_1_ exhibit impaired HSPC mobilization in response to AMD3100, a CXCR4 antagonist, while FTY720 inhibits HSPC mobilization mediated by CXCR4 antagonists but not by G-CSF [45, 102, 103]. Furthermore, FTY720 has been reported to enhance CXCR4-dependent migration and BM homing of HSPCs [47]. In our study, we demonstrated that S1P interferes with CXCR4-mediated migration when cotreated withCXCL12 by acting through CXCR4-S1P_1_ heteromers (Fig. 6F). Interestingly, pretreatment with S1P or FTY720P enhanced CXCL12-induced migration of KARPAS299 cells by downregulating surface S1P_1_, thereby alleviating S1P_1_-mediated interference of CXCR4 (Fig. 6G, H), aligning well with prior reports of reduced HSPC mobilization by FTY720 [47]. These findings suggest that G protein-biased S1P_1_ agonists, which do not induce S1P_1_ internalization, could synergistically enhance HSPC mobilization when used in combination with CXCR4 inhibitors. This dual approach could potentiate CXCR4 inhibition while maintaining responsiveness to the S1P gradient in peripheral blood.

## Conclusion

Our study demonstrates CXCR4-S1P_1_ heteromerization and reveals that the S1P/S1P_1_ axis regulates CXCR4 function by interfering with G protein association, reducing oligomerization, and suppressing CXCR4-mediated signaling and migration, without altering surface expression or ligand binding. These findings highlight the critical role of S1P_1_ in modulating CXCR4-driven immune cell trafficking and provide valuable insights into its physiological and pathological implications.

## Electronic supplementary material

Below is the link to the electronic supplementary material.

Supplementary Material 1

## Data Availability

No datasets were generated or analysed during the current study.

## Abbreviations

BRET: bioluminescence resonance energy transfer
BM: bone marrow
CXCR4: C-X-C chemokine receptor type 4
D2R: dopamine receptor D2
DMEM: Dulbecco’s modified Eagle’s medium
FBS fetal: bovine serum
FTY720P: FTY720-phosphate
GOI: gene of interest
GPCRs: G protein-coupled receptors
HSPCs: hematopoietic stem/progenitor cells
LN: lymph node
mCit: mCitrine
MFI: mean fluorescence intensity
RPMI: Roswell Park Memorial Institute
S1P: sphingosine-1-phosphate
S1P_1_: S1P receptor type 1
SLIC: sequence-and ligation-independent cloning
TM: transmembrane domain
