# Supplementary material for: Regulation of CXCR4 function by S1P1 through heteromerization
Source: Cell Commun Signal. 2025 Feb 26;23:111. doi: 10.1186/s12964-025-02099-x (PMC11863771; doi:10.1186/s12964-025-02099-x)
Supplement: Supplementary file 1 — Supplementary Material 1 [file 12964_2025_2099_MOESM1_ESM.pdf]

SUPPLEMENTARY INFORMATION

Regulation of CXCR4 function by $S1P_1$ through heteromerization

Hyun-Tae Kim[1], Jae-Yeon Jeong[2]*, Won-Ki Huh[1,2,3]*

[1]School of Biological Sciences, Seoul National University, Seoul 08826, Republic of Korea
[2]GPCR Therapeutics Inc., Gwanak-gu, Seoul 08790, Republic of Korea
[3]Institute of Microbiology, Seoul National University, Seoul 08826, Republic of Korea

*Correspondence:
Jae-Yeon Jeong
jeongjy@gpcr.co.kr (J.Y.J.)
Won-Ki Huh
wkh@snu.ac.kr (W.K.H.)

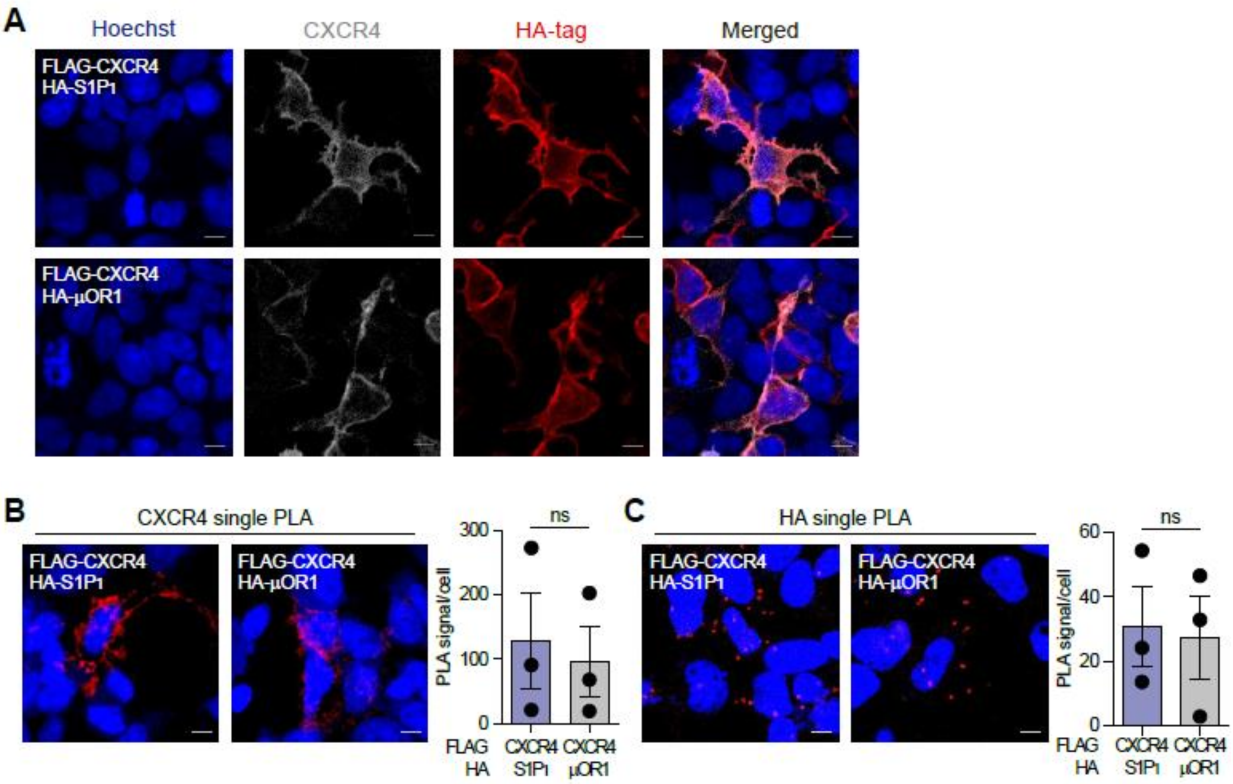

Supplementary Figure 1. Immunocytochemistry and single PLA of CXCR4, S1P$_1$, and μOR1 in HEK293A cells. (**A**) Expression of Flag-CXCR4, HA-S1P$_1$, and HA-μOR1 was assessed using immunocytochemistry following permeabilization with 0.1% Triton X-100 for 15 min. Cells were stained with anti-CXCR4 (4G10) and anti-HA-tag (C29F4) antibodies to visualize protein localization. Cells were visualized using Hoechst 33342 (blue) for nuclei, AF647 (gray) for CXCR4, and AF568 (red) for HA-tagged GPCRs (S1P$_1$ and μOR1). Scale bars: 10 μm. (**B, C**) Single PLA was performed under non-permeabilized conditions to detect homomer formation of CXCR4 (B) or HA-tagged S1P$_1$ or μOR1 (C) on the surface using anti-CXCR4 (4G10) and anti-HA-tag (C29F4) antibodies. PLA signals were shown in red and cells were visualized using Hoechst 33342 (blue) for nuclei. Images are representative of three independent experiments. Data represent the mean ± SEM of n = 3 independent experiments. Statistical significance was tested using two-tailed Student's *t*-test (B, C). ns, not significant. Scale bars: 10 μm.

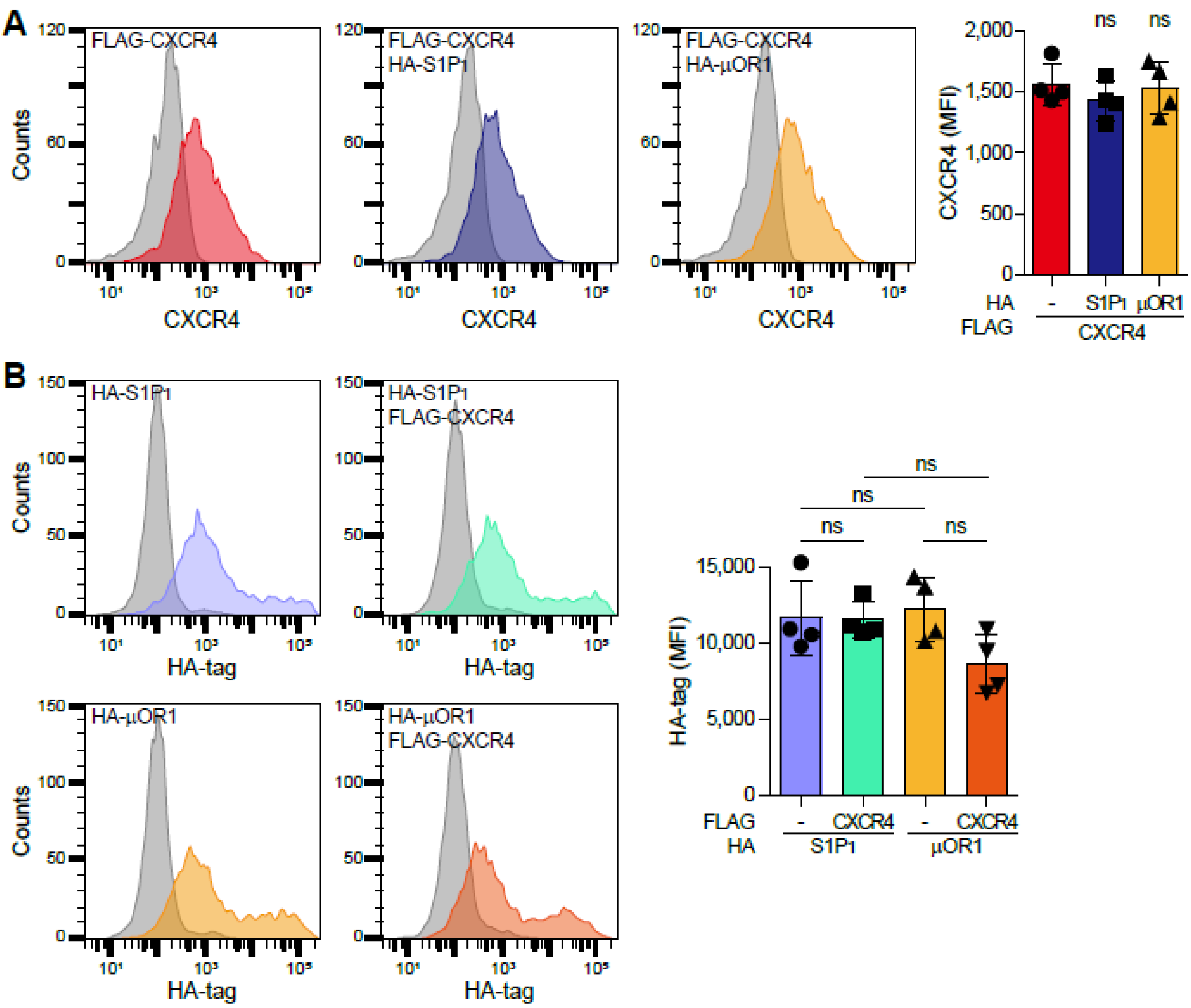

Supplementary Figure 2. Effect of GPCR coexpression on surface expression of partner GPCRs. (**A**) Surface expression of CXCR4 was analyzed using flow cytometry in HEK293A cells. Anti-CXCR4 (Ulocuplumab) antibody was used to detect CXCR4 in cells expressing Flag-CXCR4 alone (red) or together with HA-$S1P_1$ (blue) or HA-µOR1 (yellow). (**B**) Surface expression of $S1P_1$ and µOR1 was analyzed using flow cytometry in HEK293A cells. Anti-HA-tag (C29F4) antibody was used to detect $S1P_1$ and µOR1 in cells expressing HA-$S1P_1$ alone (light blue) or together with CXCR4 (light green) or HA-µOR1 alone (yellow) or together with CXCR4 (orange). Gray histograms represented staining with IgG control antibody. Representative histograms of four independent experiments are shown. Data represent the mean ± SD of n = 4 independent experiments. Statistical significance was tested using one-way ANOVA followed by Dunnett's post-hoc test (A) and Tukey's post-hoc test (B) at the indicated conditions. ns, not significant.

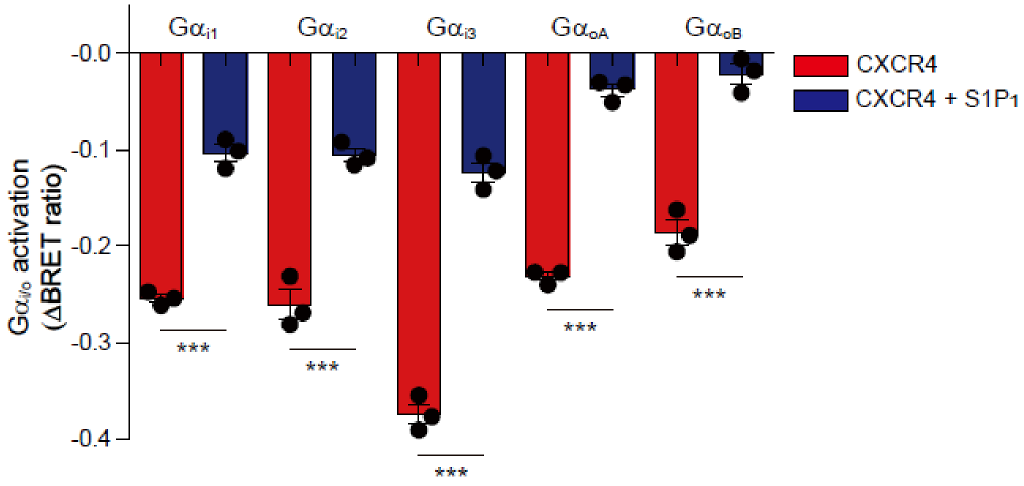

Supplementary Figure 3. Inhibition of CXCR4-mediated $G\alpha_{i/o}$ activation by $S1P_1$. CXCL12 (10 nM)-induced G protein activation of five $G\alpha_{i/o}$ proteins was measured in HEK293A cells expressing CXCR4 alone or together with $S1P_1$. Data represent the mean ± SEM of n = 3 independent experiments. Statistical significance was tested using two-tailed Student's *t*-test. ***$P < 0.001$.

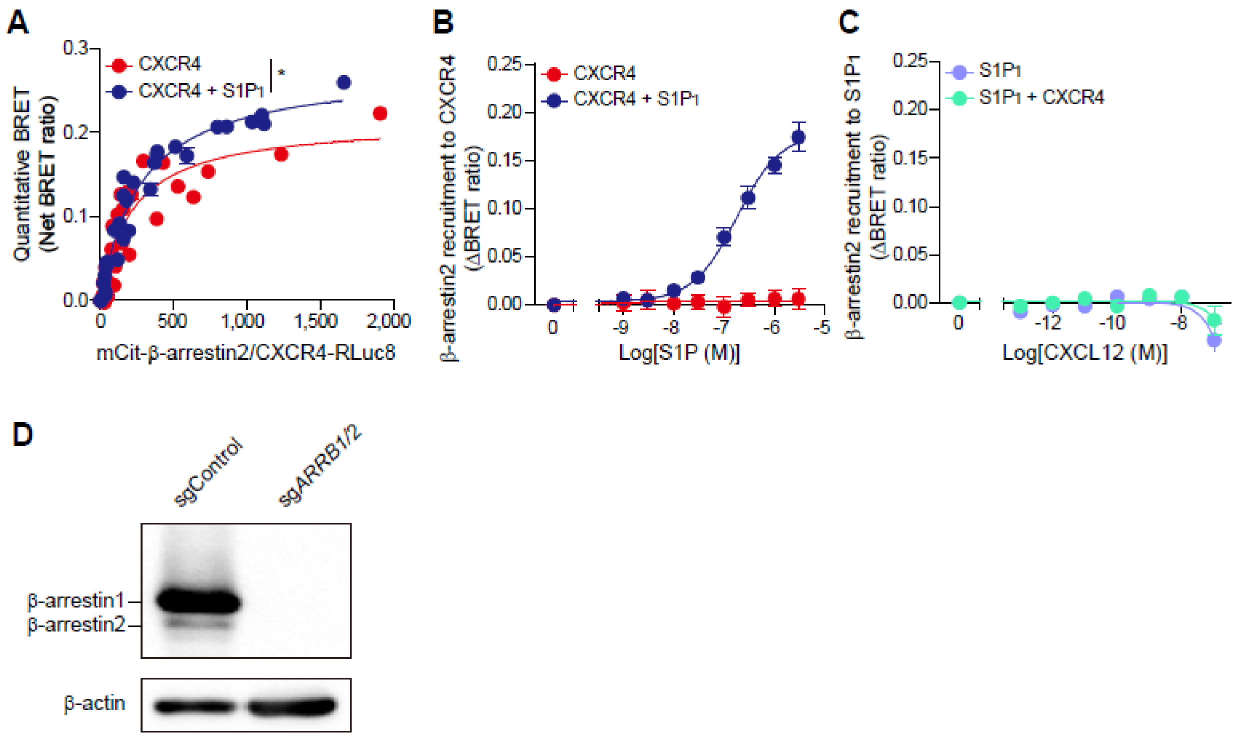

Supplementary Figure 4. CXCL12- or S1P-induced β-arrestin2 recruitment to CXCR4 or $S1P_1$ and validation of *ARRB1/2* knockout in HEK293A cells. (**A**) Quantitative BRET assay was performed in HEK293A cells between mCit- β-arrestin2 and CXCR4-Rluc8 in the absence or presence of $S1P_1$ and/or CXCL12 (10 nM). The curves were fitted using a non-linear regression equation and represent three independent experiments. (**B**) S1P-induced β-arrestin2 recruitment to CXCR4 was measured in HEK293A cells expressing CXCR4-Rluc8 alone or together with $S1P_1$. (**C**) CXCL12-induced β-arrestin2 recruitment to $S1P_1$ was measured in HEK293A cells expressing $S1P_1$-Rluc8 alone or together with CXCR4. (**D**) Western blotting was performed to validate deletion of β-arrestin1/2 in control and β-arrestin1/2-deficient HEK293A cells. Data represent the mean ± SEM of n = 4 independent experiments. Statistical significance was tested using two-tailed Student's *t*-test for $BRET_{max}$ (A). *$P < 0.05$.

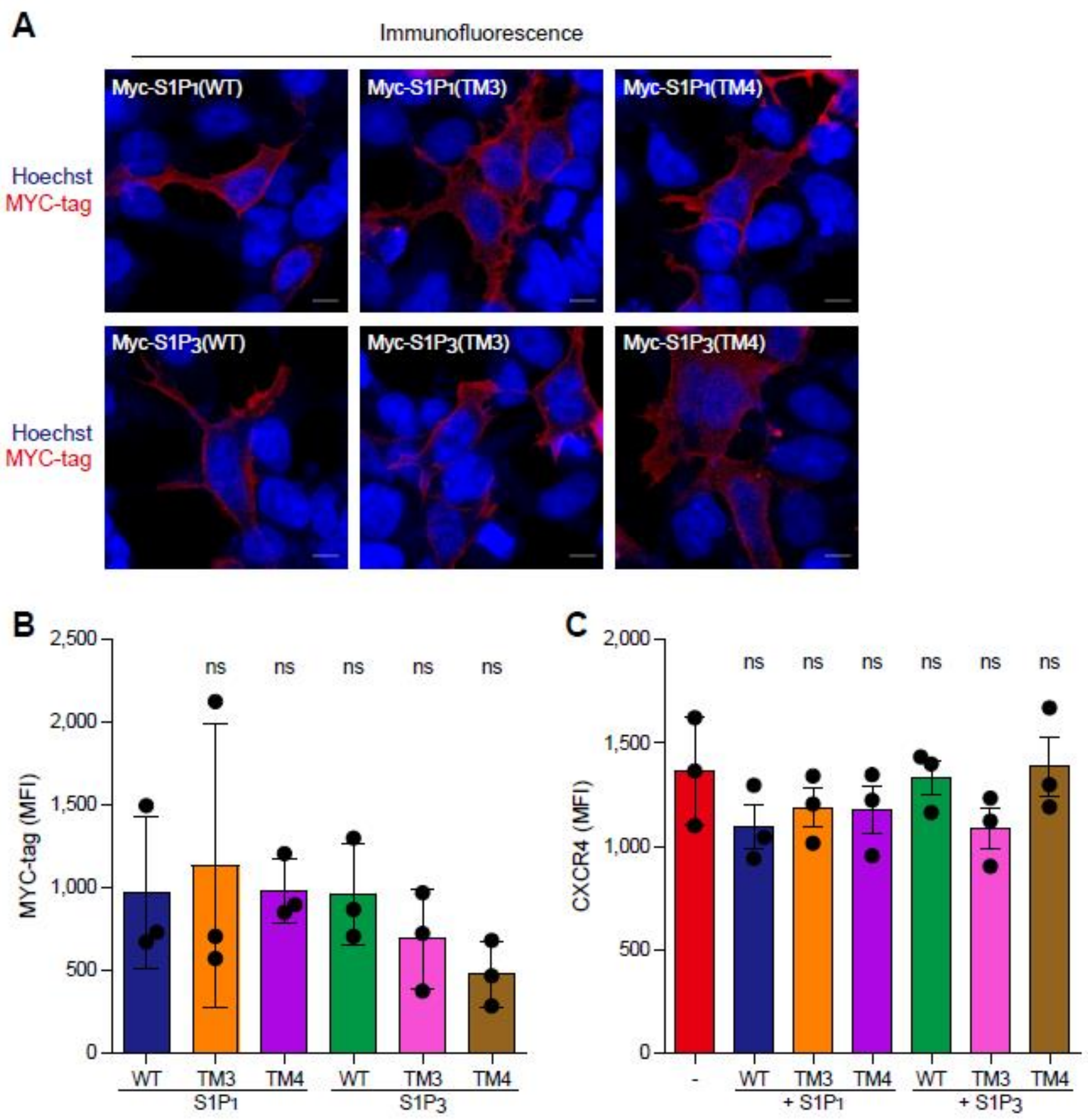

Supplementary Figure 5. Surface expression of domain-swapped $S1P_1/S1P_3$ mutants and CXCR4 in HEK293A cells. (**A**) Expression of $S1P_1$, $S1P_3$, and their domain-swapped mutants was detected by immunocytochemistry using anti-MYC-tag (9B11) antibody. Cells were visualized using Hoechst 33342 (blue) for nuclei and AF647 (red) for MYC-tagged GPCRs. (**B**) Surface expression of MYC-tagged $S1P_1$, $S1P_3$, and their domain-swapped mutants was analyzed using flow cytometry in HEK293A cells transfected with equal amounts of plasmids encoding each GPCR. Anti-MYC-tag (9B11) antibody was used to detect MYC-tagged GPCRs. (**C**) Surface expression of CXCR4 was analyzed using flow cytometry in HEK293A cells transfected Flag-CXCR4 alone or together with $S1P_1$, $S1P_3$, and their domain-swapped mutants. Anti-CXCR4 (Ulocuplumab) antibody was used to detect CXCR4. Data represent the mean ± SD of n = 3 independent experiments. Statistical significance was tested using one-way ANOVA followed by Dunnett's post-hoc test. ns, not significant.

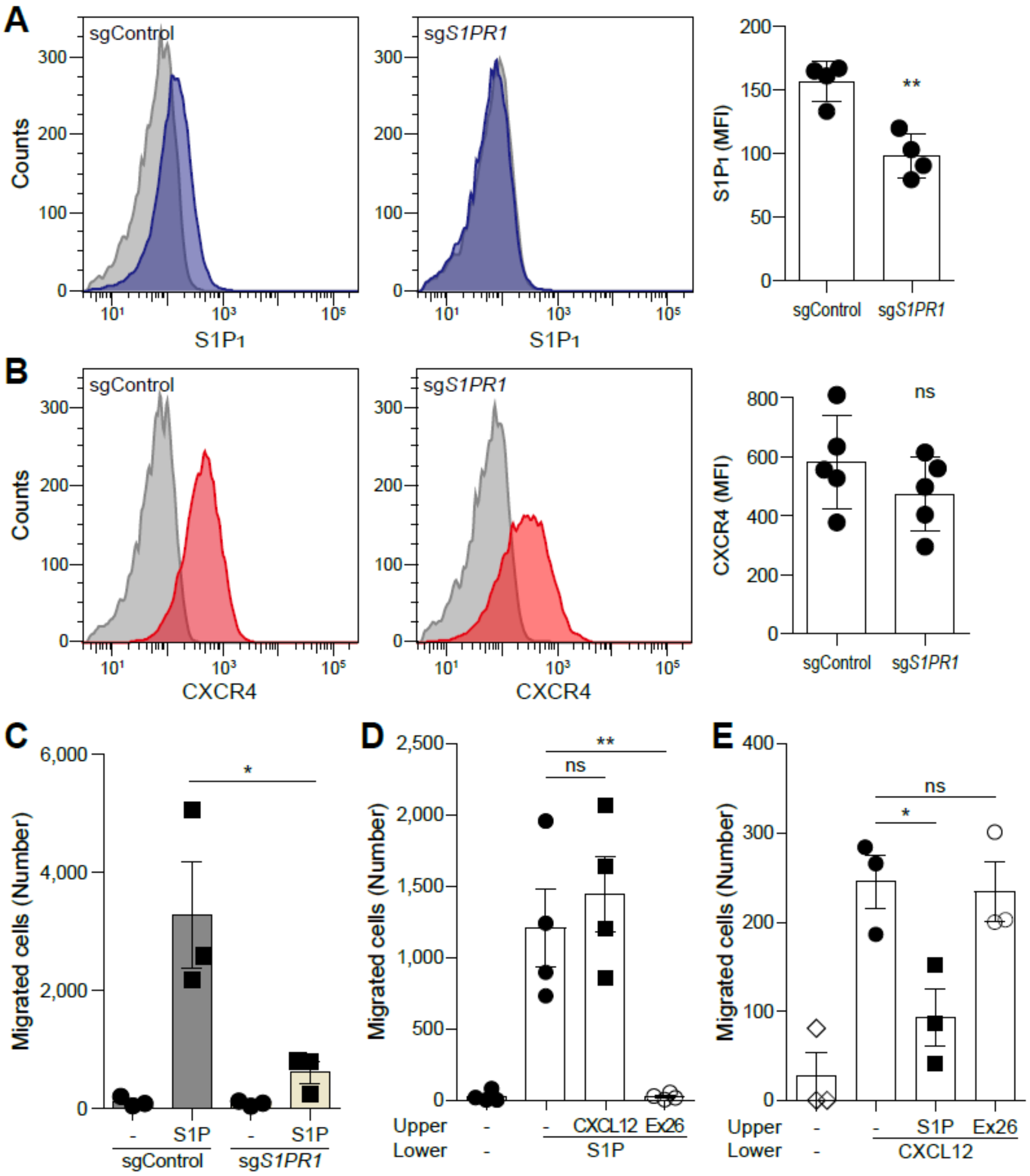

Supplementary Figure 6. Validation of *S1PR1* knockout in KARPASS299 cells and S1P-induced cell migration. (**A, B**) Surface expression of $S1P_1$ and CXCR4 was measured in control and $S1P_1$-deficient KARPAS299 cells. Anti-$S1P_1$ (#218713) antibody was used to detect $S1P_1$ (A) and anti-CXCR4 (Ulocuplumab) antibody was used to detect CXCR4 (B). (**C**) Transwell migration assay was performed to measure S1P (10 nM)-induced migration of control and $S1P_1$-deficient KARPAS299 cells. (**D, E**) Transwell migration assay was performed in KARPAS299 cells to measure the effects of CXCL12 (30 nM) and Ex26 (10 μM) on S1P (10 nM)-induced cell migration (D) and the effects of S1P (10 nM) and Ex26 (10 μM) on CXCL12 (30 nM)-induced cell migration (E). Gray histograms represent staining with IgG control antibody, and representative histograms of five independent experiments are shown (A, B). Data represent the mean ± SD of n = 4 to 5 independent experiments (A, B) and the mean ± SEM of n = 3 to 4 independent experiments (C-E). Statistical significance was tested using two-tailed Student's *t*-test (A-C) and one-way ANOVA followed by Dunnett's post-hoc test (D, E). *$P < 0.05$; **$P < 0.01$; ns, not significant.

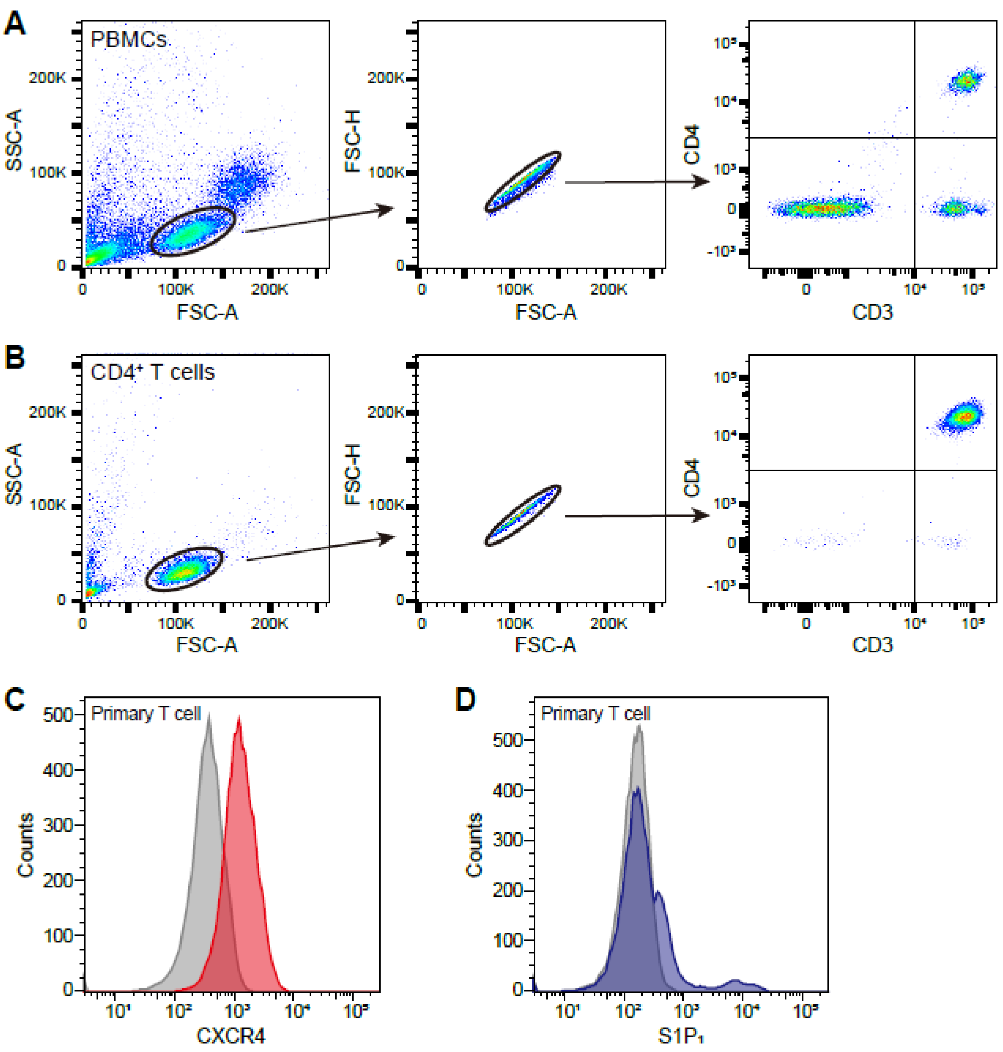

Supplementary Figure 7. Gating strategy and surface expression of CXCR4 and $S1P_1$ in primary T cells. (**A, B**) Gating strategy for PBMCs (A) and CD4+ T cells (B). The population and expression characteristics of PBMCs and CD4+ T cells were compared before (A) and after (B) isolation using anti-CD3 (UCHT1) and anti-CD4 (RPA-T4) antibodies. (**C**) Surface expression of CXCR4 was measured in primary T cells using anti-CXCR4 (4G10) antibody. (**D**) Surface expression of $S1P_1$ was measured in primary T cells using anti-$S1P_1$ (#218713) antibody. Gray histograms represent staining with IgG control antibody. Representative histograms of six independent experiments are shown.
